# Combined Lingzhi Huang capsules and Zeng Jian health tonic accelerates skin wound healing via BMP5-mediated inhibition of ferroptosis

**DOI:** 10.3389/fimmu.2026.1818752

**Published:** 2026-06-02

**Authors:** Li Liu, Ying Luo, Xiaoliang Lin, Liang Chen, Zumeng Xia, Hongzhang Chen, Hongjue Wang, Ruo-Pan Huang, Ming Liang

**Affiliations:** 1 Infinitus (China) Company Ltd., Guangzhou, China; 2 Department of Biomedical Engineering, School of Materials Science and Engineering, South China University of Technology, Guangzhou, Guangdong, China; 3 South China Biochip Research Center, Guangzhou, Guangdong, China; 4 Affiliated Cancer Hospital and Institute of Guangzhou Medical University, Guangzhou, Guangdong, China; 5 Guangdong Provincial Hospital of Chinese Medicine, Guangzhou, Guangdong, China

**Keywords:** Bmp5, ferroptosis, inflammation, Lingzhi Huang capsules, oxidative Stress, wound healing, Zeng Jian health tonic

## Abstract

**Introduction:**

Lingzhi Huang capsules (LZH) and Zeng Jian Health Tonic (ZJ) are traditional Chinese medicinal formulations enriched in polysaccharides and antioxidant components. They have been traditionally used to enhance immunity function and reduce inflammation, promote tissue repair. However, their therapeutic efficacy and underlying mechanisms in skin wound healing have not been fully elucidated. Guided by the Traditional Chinese Medicine principle of “treating the same disease with different therapeutic strategies,” this study aimed to evaluate the effects of LZH and ZJ, administered individually and in combination, on skin wound healing and to elucidate the associated molecular mechanisms.

**Methods:**

The chemical constituents of LZH and ZJ were characterized using liquid chromatography-tandem mass spectrometry (LC-MS/MS). A full‐thickness skin wound model in mice was employed to assess therapeutic effects through histopathological analysis, immunofluorescence, Western blotting, and protein antibody array analysis. In vitro, human umbilical vein endothelial cells (HUVECs) were used to investigate angiogenesis, inflammation, oxidative stress, and ferroptosis. Network pharmacology integrated with protein array profiling was applied to identify key targets and signaling pathways involved in the combined treatment, which were subsequently validated through mechanistic experiments.

**Results:**

LZH and ZJ significantly accelerated wound closure, enhanced collagen deposition, and promoted angiogenesis, with the combined treatment exhibiting the most pronounced effects. In HUVECs, LZH + ZJ markedly enhanced cell proliferation, migration, and tube formation; increased CD31 and VEGF expression; reduced reactive oxygen species (ROS) levels; elevated SOD and GSH; decreased MDA levels, and alleviated mitochondrial damage. These effects were accompanied by upregulation of GPX4, SLC7A11, and FTH1. Integrated network pharmacology and protein array analyses revealed enrichment of pathways associated with angiogenesis, inflammation, and ferroptosis, and identified BMP5 as a central regulatory target. Mechanistic validation demonstrated that BMP5 overexpression recapitulated the anti-ferroptotic effects of LZH + ZJ, whereas BMP5 knockdown abolished these protective effects.

**Conclusion:**

Combined administration of LZH and ZJ promotes skin wound healing more effectively than either treatment alone by suppressing ferroptosis, inflammation, and oxidative stress through BMP5-mediated signaling. These findings provide mechanistic support for the traditional therapeutic rational and suggest a promising strategy for enhancing wound healing.

## 1 Introduction

Skin wound healing is a well-orchestrated biological process involving overlapping stages including hemostasis, inflammation, cell migration and proliferation, extracellular matrix deposition, and tissue remodeling, which ultimately restores skin barrier integrity [1, 2]. Effective repair depends on the precise temporal and spatial coordination of cytokines, growth factors, and diverse cell populations [3]. The balance between inflammation and oxidative stress is particularly critical. Dysregulated inflammation and excessive reactive oxygen species (ROS) can impair angiogenesis, delay re-epithelialization, and contribute to chronic or non-healing wounds [4, 5]. Ferroptosis is a regulated cell death pathway characterized by iron-dependent lipid peroxidation and ROS overaccumulation, which causes cellular dysfunction [6]. Excessive ROS generated during local inflammatory responses can disrupt intracellular iron homeostasis, leading to labile iron accumulation, which in turn promotes lipid peroxidation and triggers ferroptosis. Ferroptosis directly damages key wound-healing cells, including keratinocytes, fibroblasts, and endothelial cells, thereby impairing re-epithelialization, inhibiting extracellular matrix synthesis and deposition, and suppressing angiogenesis[7, 8]. Notably, ferroptosis-mediated cellular injury can disrupt the normal progression of wound healing, especially in non-diabetic conditions, ultimately leading to delayed healing or chronic wound formation. Inhibition of lipid peroxidation has been shown to reduce intracellular oxidative damage, promote cell proliferation, and restore cellular function, suggesting that targeting ferroptosis may represent a promising therapeutic strategy for wound healing[9, 10]. However, impaired and delayed wound healing remains a major global clinical burden with limited effective treatments. Thus, novel therapeutic strategies for improved wound management are urgently needed.

Traditional Chinese Medicine (TCM), with a 5,000-year documented history, provides holistic, multi-component, and multi-target therapeutic approaches [11, 12]. For wound repair, herbal formulas are particularly promising, as they may simultaneously modulate inflammation, suppress oxidative stress, promote angiogenesis, support tissue regeneration, and optimize the wound microenvironment [13]. Accumulating experimental evidence supports this potential [14, 15]. For example, Ganoderma lucidum spore oil, has been shown to accelerate cutaneous wound closure by activating of the TRPV1/TGF-β/SMAD pathway [16]. In addition, the natural pentacyclic triterpene lupeol has been reported to enhance wound repair under hyperglycemic conditions [17]. Polysaccharides from Tremella fuciformis promote wound healing by enhancing re-epithelialization and collagen deposition, while also providing antioxidant and moisturizing effects that support skin regeneration [18]. Astragalus-derived polysaccharide preparations restore inflammatory homeostasis and stimulate angiogenesis in cutaneous wound models [19, 20]. Polysaccharides from edible mushrooms such as Lentinula edodes exhibit potent immunomodulatory and antioxidant activities *in vitro* and *in vivo*, supporting their utility in tissue repair [21]. Collectively, these findings underscore the therapeutic potential of multi-component herbal formulations for targeting the complex pathologies underlying impaired wound healing.

Lingzhi Huang capsules (LZH), which contain Ganoderma lucidum, *Atractylodis macrocephalae Rhizoma, Polygonati Rhizoma, Lycii Fructus, Coicis Semen, Poria, Ophiopogonis Radix, Cuscutae Semen, Schisandrae Fructus and Glycyrrhizae Radix*, and Zeng Jian Health Tonic (ZJ), composed of *Lentinula edodes, Poria, Tremella fuciformis, Cassiae Semen, Flammulina velutipes, Lycii Fructus, Chrysanthemi Flos and Mori Fructus*, are both rich in polysaccharides, triterpenoids, antioxidants and immunomodulatory compounds. These bioactive constituents have been reported to regulate key processes involved in wound healing, including inflammatory responses, angiogenesis, and oxidative stress [22, 23]. Notably, accumulating clinical and translational evidence supports the therapeutic relevance of polysaccharide-rich TCM formulations in wound repair. Polysaccharides derived from Ganoderma lucidum have been shown to accelerate wound healing and improve tissue perfusion in diabetic wound models, while Astragalus polysaccharides are associated with enhanced tissue regeneration through modulation of inflammatory responses [19, 24]. Beyond single-compound studies, bioactive polysaccharides from medicinal fungi such as Ganoderma lucidum, Tremella fuciformis, and Lentinula edodes have been implicated in promoting angiogenesis, alleviating oxidative stress, and facilitating tissue regeneration in clinically relevant settings [25, 26]. Clinically, polysaccharide-based TCM preparations have demonstrated beneficial effects in patients with chronic wounds and diabetic ulcers, including accelerated wound closure, improved granulation tissue formation, and enhanced angiogenesis [27, 28]. Despite these pharmacological and clinical findings, the specific effects of LZH and ZJ on cutaneous wound healing remain unclear. Although the two formulations share overlapping bioactivities, their distinct phytochemical profiles suggest potentially complementary or synergistic mechanisms of action. LZH is enriched in triterpenoids and polysaccharides derived from Ganoderma lucidum, Poria cocos, and related medicinal herbs, which are associated with immunomodulatory and anti-inflammatory effects. In contrast, ZJ contains abundant polysaccharides and antioxidant constituents from Lentinula edodes, Tremella fuciformis, and other botanical sources, which are linked to ROS scavenging, redox homeostasis, and angiogenesis. Based on these complementary or synergistic bioactive characteristics, we hypothesize that the combination of LZH and ZJ may exert synergistic effects by simultaneously targeting multiple key processes involved in wound healing, including inflammation, oxidative stress, and angiogenesis. This hypothesis aligns with the traditional Chinese medicine principle of “treating the same disease with different therapeutic strategies” and suggests that combined therapy may achieve superior therapeutic outcomes compared with monotherapy. Elucidating the potential synergistic interactions between LZH and ZJ may provide valuable insights into multi-component herbal strategies for enhancing wound repair.

Building on these complementary bioactive profiles, this study aims to systematically evaluate the effects of LZH and ZJ alone and in combination on cutaneous wound healing and elucidate their underlying mechanisms. Importantly, despite their widespread traditional use, their effects on wound repair remain to be systematically characterized.

## 2 Materials and methods

### 2.1 LC-MS/MS analysis

The constituents of LZH and ZJ were analyzed using UFLC-Q-TOF-MS/MS. Chromatographic separation was performed on a BEH C8 column (2.1 × 100 mm, 1.7 μm) and an HSS T3 column (2.1 × 100 mm, 1.8 μm) at 40 °C with mobile phase A (0.1% formic acid in water) and mobile phase B (0.1% formic acid in acetonitrile). The flow rate was set at 0.35 mL/min, and the injection volume was 5 μL. Data were acquired in both positive and negative ionization modes, and total ion chromatograms (TICs) were generated for analysis.

### 2.2 Animal wound healing model

Male C57BL/6 mice (8–10 weeks old, weighing 20–22 g) were purchased from Ruige Biological Technology Co., Ltd. The mice were housed in a specific pathogen - free (SPF) environment with a 12 h light/dark cycle, constant temperature (22 ± 2 °C), and constant humidity (50 ± 5%). They were provided with standard laboratory food and water ad libitum. All experimental procedures were approved by the Ethics Committee of Guangzhou Seyotin Biotechnology Co., LTD (SYT2025043).

To establish the skin wound model, mice were anesthetized by intraperitoneal injection of pentobarbital sodium (50 mg/kg). The dorsal hair was shaved with an electric clipper, and the skin was disinfected with 75% ethanol. A full-thickness circular wound (10 mm in diameter) was created on the dorsal skin using a sterile biopsy punch. Mice were randomly assigned to five groups using a random number table (n = 10 per group): control group, positive control group (epidermal growth factor, EGF), LZH group (0.25 g/kg), ZJ group (3.9 g/kg), and LZH + ZJ group (0.25 g/kg + 3.9 g/kg). Investigators involved in wound evaluation, image acquisition, and data analysis were blinded to group allocation, with treatment groups coded prior to the experiment and all assessments performed without knowledge of treatment identities. The sample size was determined based on preliminary experiments and previous studies to ensure adequate statistical power. The doses of LZH and ZJ were determined based on clinical dosage, body surface area conversion, and our preliminary experiments. Specifically, two doses of LZH (0.125 and 0.25 g/kg) and two doses of ZJ (1.95 and 3.9 g/kg) were evaluated. In addition, different combination ratios of LZH and ZJ (1:1, 2:1, and 1:2) were assessed to identify the optimal formulation, as shown in **Supplementary Figures S1**, **S2**. Based on these results, 0.25 g/kg for LZH and 3.9 g/kg for ZJ at a 1:1 ratio were selected for subsequent experiments. All treatments were administered once daily by oral gavage, while recombinant human EGF was topically applied to the wound area once daily [29, 30] for a total duration of 14 days. Wound closure was documented on days 0, 3, 7, and 14. Skin tissues were harvested on days 7 and 14 for subsequent analyses.

### 2.3 Cell culture and treatment

Human Umbilical Vein Endothelial Cells (HUVECs) were purchased from Wuhan Pricella Biotechnology Co., Ltd. The cells were cultured in DMEM supplemented with 10% FBS and 1% penicillin-streptomycin in a humidified incubator at 37 °C with 5% CO_2_. HUVECs were treated with different concentrations of LZH, ZJ and LZH + ZJ for durations specified according to experimental requirements. To establish an *in vitro* model, HUVECs were exposed to 100 μg/mL lipopolysaccharide (LPS) for 24 h [31] (**Supplementary Figure 6C**).

### 2.4 Cell counting Kit-8 assay

The cell viability of HUVEC was determined using Cell Counting Kit-8 (CCK-8, Beyotime, China). Cells were seeded into 96-well plates and treated with different concentrations of the drug. Subsequently, 10 µl of CCK-8 reagent was added to each well and incubated at 37 °C for 2 h. The absorbance was then measured at 450 nm using a microplate reader.

### 2.5 Cell proliferation assay

Cell proliferation of HUVECs was assessed using an EdU assay. Cells were treated under the same conditions and incubated with 10 µM EdU reagent. After fixation and permeabilization, cells were processed using a commercial EdU-Click 488 kit following the manufacturer’s protocol. Nuclei were counterstained with Hoechst 33342, and fluorescence images were acquired under a fluorescence microscope.

### 2.6 Transwell migration assay

The Transwell assay was performed to evaluate cell migration. Cell suspensions in serum-free medium were seeded into the upper chamber of the Transwell insert, while the lower chamber was filled with 600 μL of culture medium supplemented with 10% FBS. After 24 h of incubation, non-migrated cells on the upper surface of the membrane were carefully removed with a cotton swab. The migrated cells on the lower surface were fixed for 30 min and stained with crystal violet for 10 min. Cells were then observed under an optical microscope, and the number of cells was quantified using ImageJ.

### 2.7 Tube formation assay

Matrigel was thawed on ice and added to a 96 - well plate. The plate was incubated at 37 °C for 30 min to allow the Matrigel to solidify. Cell suspensions were seeded onto the gelled matrix and incubated for 6 h. Tube formation was observed under a light microscope and counted using ImageJ.

### 2.8 Quantitative real-time PCR

Total RNA was isolated from skin tissues or cultured cells using TRIzol reagent (Seyotin, Guangzhou, China). cDNA synthesis was performed using a reverse transcription kit (Seyotin, Guangzhou, China). qPCR was conducted using SYBR Green Master Mix on an ABI 7500 system. GAPDH was used as the internal control. Relative expression levels were calculated using 2^−ΔΔCt^ method. Primer sequences are listed in **Supplementary Table 1**.

### 2.9 Western blotting

Proteins were extracted from tissues or cells with RIPA buffer containing protease inhibitors. The protein concentration was determined using a BCA protein assay kit (Seyotin, Guangzhou, China). Equal amounts of protein were separated by sodium dodecyl sulfate - polyacrylamide gel electrophoresis (SDS - PAGE) and transferred to polyvinylidene fluoride (PVDF) membranes. The membranes were blocked with 5% skimmed milk for 1 h at room temperature and then incubated with primary antibodies against CD31, VEGF, GPX4, SLC7A11, FTH1, TNF-α, IL-6, IL-1α and GAPDH overnight at 4 °C. The next day, the membranes were incubated with HRP - conjugated secondary antibodies for 2 h at room temperature. The protein bands were visualized using an enhanced chemiluminescence (ECL) detection kit, and the gray value of each band was analyzed using ImageJ software.

### 2.10 Immunofluorescence staining

Tissue and cell samples were fixed with 4% paraformaldehyde, permeabilized with 0.3% Triton X-100, and blocked with 5% bovine serum albumin for 2 h at room temperature. The samples were then incubated overnight at 4 °C with primary antibodies against α-SMA, CD31, VEGF, GPX4, and BMP5. After washing, secondary fluorophore-conjugated antibodies were applied. Nuclei were counterstained with DAPI, and fluorescent images were captured using a fluorescence microscope.

### 2.11 Flow cytometry

Cell culture supernatants from different treatment groups were collected and incubated with 10 μM DCFH-DA probe at 37 °C for 30 min in the dark. The production of reactive oxygen species (ROS) was then detected using a flow cytometer.

### 2.12 Enzyme-linked immunosorbent assay

The levels of SOD, GSH, and MDA in cell culture supernatants and tissue were detected using Elisa Kits following the manufacturer’s instructions.

### 2.13 Transmission electron microscopy

Cells were fixed with 2.5% glutaraldehyde at 4 °C overnight, post-fixed with 1% osmium tetroxide, dehydrated through a graded ethanol series, embedded in epoxy resin, and sectioned into ultrathin slices. The sections were stained with uranyl acetate followed by lead citrate, and examined under a transmission electron microscope (Hitachi) with appropriate magnification and focus to assess mitochondrial morphology.

### 2.14 Network pharmacology analysis

The active components of LZH and ZJ were collected from the Traditional Chinese Medicine Systems Pharmacology Database and Analysis Platform (TCMSP, accessed on May 2025), BATMAN-TCM (score cutoff of 0.84 [LR =0.88], an adjusted P-value ≤ 0.05, and a druggability score ≥ 0.1), HERB and mass spectrometry analysis results, based on the criteria of oral bioavailability (OB ≥ 30%) and drug-likeness (DL ≥ 0.18). The potential targets of these active components were predicted using the SwissTargetPrediction database (probability ≥ 0.1) and TCMSP. The wound healing - related targets were obtained from the GeneCards (version 5.0) and OMIM databases. A Venn diagram was created using the jvenn online website. The herb-compound-target network was built utilizing Cytoscape 3.7.1. PPI network was obtained by the STRING database (version 11.5, confidence score ≥ 0.4), then visualized using Cytoscape software. Gene Ontology (GO) and Kyoto Encyclopedia of Genes and Genomes (KEGG) enrichment analyses were performed using DAVID 6.8.

### 2.15 Protein antibody array

Protein antibody array were performed following standard procedures. Microarray chips were equilibrated, thoroughly dried, and subsequently blocked with 100 µL of sample diluent for 1 h. Standards or twofold-diluted samples were then applied and incubated overnight at 4 °C. Following incubation, chips were washed on a Wellwash Versa system, with each well washed 10 times with 1× Wash Buffer I followed by 6 washes with 1× Wash Buffer II. Detection antibodies were added and incubated for 2 h, after which Cy3-streptavidin was applied for 1 h under light-protected conditions, followed by the same washing procedure. Fluorescence signals were acquired using an InnoScan 300 scanner and processed for data analysis.

### 2.16 Co-immunoprecipitation assay

To investigate protein–protein interactions between BMP5 and ferroptosis-related proteins, Co-IP assays were performed in HEK293T cells. Cells were collected and lysed in lysis buffer containing protease inhibitors. Equal amounts of cell lysates were first pre-cleared with Protein A/G agarose beads, and then incubated with anti-HA or anti-Flag antibodies overnight at 4 °C. Subsequently, Protein A/G agarose beads were added and incubated for 4 h at 4 °C. After extensive washing, the immunocomplexes were eluted with elution buffer and analyzed by Western blotting.

### 2.17 Statistical analysis

All experiments were independently repeated at least three times. Data are presented as mean ± SD. Statistical analysis was performed using GraphPad Prism 9.5 software. Comparisons between multiple groups were conducted using one-way analysis of variance (ANOVA) or two-way analysis of variance (ANOVA) followed by Tukey’s *post hoc* test, and *P* < 0.05 was considered statistically significant.

## 3 Results

### 3.1 Chemical components of LZH and ZJ

**Figure 1:**
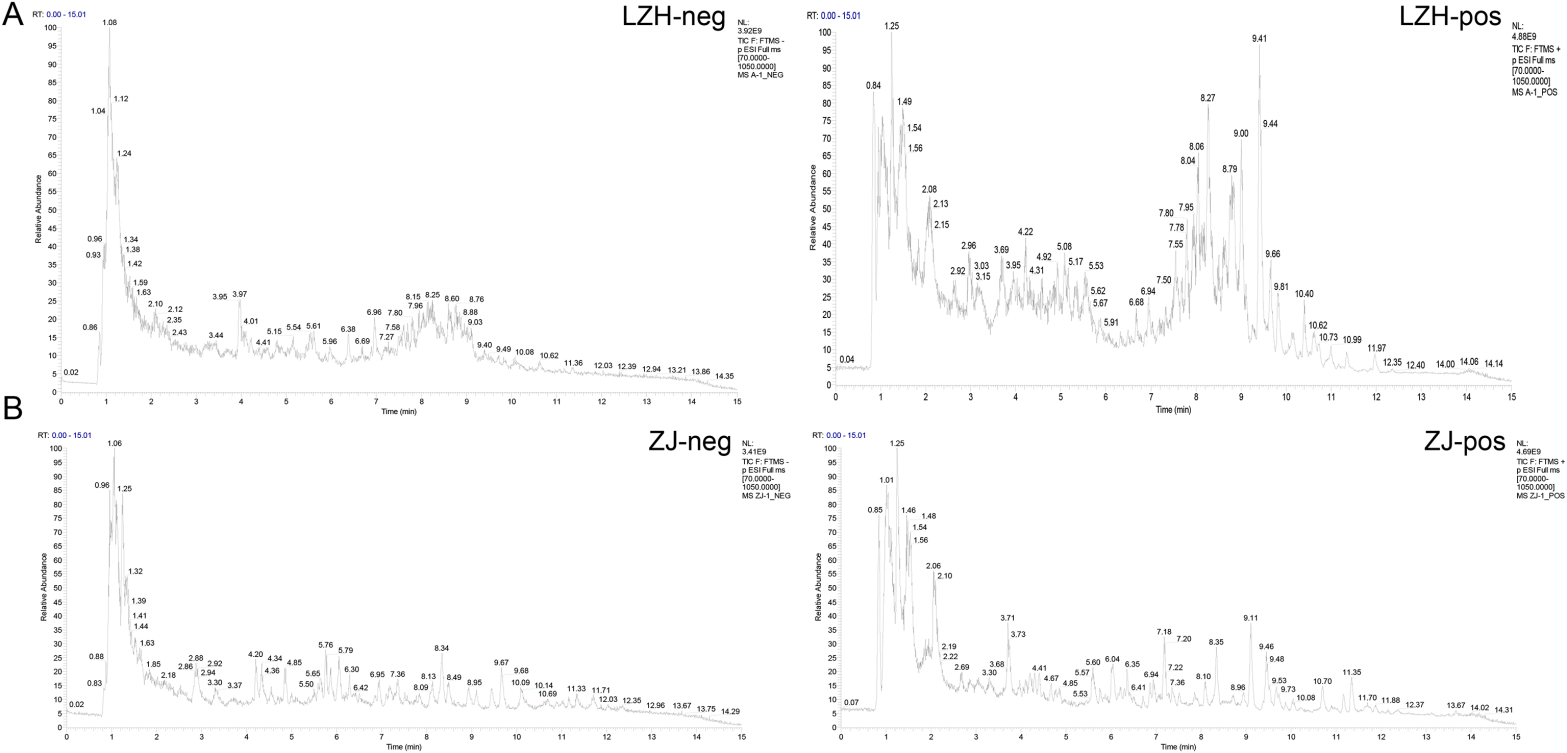
Chemical components of LZH and ZJ. **(A)** Total ion chromatograms (TIC) of LZH in positive ion mode and negative ion mode. **(B)** Total ion chromatograms (TIC) of ZJ in positive ion mode and negative ion mode.

The chemical constituents of LZH and ZJ were profiled using LC-MS/MS. The resulting total ion chromatograms in both positive and negative ion modes are shown in **Figure 1**. As shown in **Supplementary Tables S1**, **S2**, a total of 89 chemical constituents were identified in LZH, primarily including flavonoids, carboxylic acids and derivatives, organooxygen compounds, fatty acyls, isoflavonoids, and other classes of compounds. In ZJ, 80 chemical constituents were identified, mainly comprising flavonoids, carboxylic acids and derivatives, organooxygen compounds, fatty acyls, prenol lipids, and other types of compounds.

### 3.2 Effect of LZH, ZJ, and LZH+ZJ on wound healing in mice

**Figure 2:**
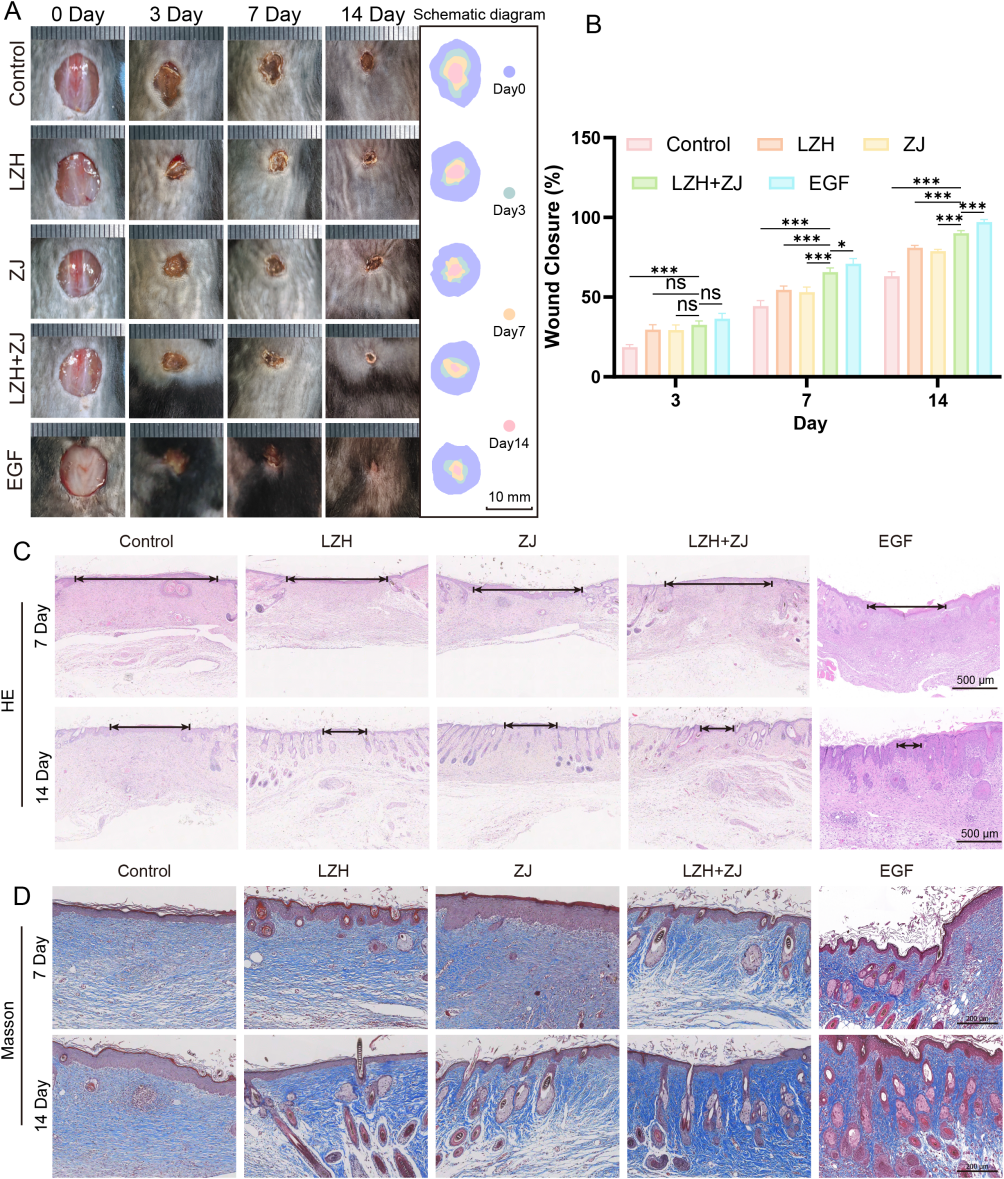
Effect of LZH, ZJ, and LZH+ZJ on wound healing in mice. **(A)** Representative wound photographs and mode patterns from control, LZH, ZJ, and LZH+ZJ groups on days 0, 3, 7, and 14. **(B)** The wound closure was quantified by calculating the percentage of the wound area relative to that measured on day 0. (n = 5). **(C)** Representative H&E staining images of wound tissues on days 7 and 14. Scale bar = 500 μm. **(D)** Representative images of Masson’s trichrome staining of skin wound tissues on day 7 and day 14. Scale bar = 200 μm. Data is presented as mean ± SD. *P*-values are calculated using two-way analysis of variance (ANOVA) for multiple group comparisons. ^*^P<0.05, ^**^P<0.01, ^***^P<0.001, ns, not significant.

Wound integrity is a critical indicator for evaluating wound healing progression. In this study, to investigate the therapeutic potential of LZH, ZJ and LZH+ZJ in promoting cutaneous wound repair in mice, representative wound images and mode patterns were obtained on days 0, 3, 7, and 14. Compared with the control group or monotherapy, the LZH + ZJ combination group showed a significantly reduced wound area and demonstrated superiority over monotherapy at day 14. Notably, on day 14, the EGF group exhibited a higher wound healing rate than the combination treatment group (**Figures 2A, B**). Histological analysis using H&E staining revealed a decrease in wound area and partial re-epithelialization in all treatment groups by day 7. These improvements became more prominent by day 14, particularly in mice treated with LZH+ZJ and EGF (**Figure 2C**). Masson’s trichrome staining demonstrated enhanced collagen deposition at day 14 compared with day 7, with the LZH+ZJ and EGF group exhibiting the most substantial collagen accumulation (**Figure 2D**).

Immunofluorescence analysis showed increased expression of α-SMA, CD31, and COL-1 in all treatment groups at day 14 relative to day 7, with the strongest signals detected in the LZH+ZJ and EGF groups (**Supplementary Figures 3A–C**). Furthermore, western blot analysis indicated elevated levels of pro-inflammatory cytokines TNF-α, IL-6, and IL-1α in the control group. These cytokines were reduced by day 7 in all treated groups and further declined by day 14, with the most pronounced effects observed in the LZH+ZJ and EGF groups (**Supplementary Figures 4A, B**). Serum cytokine antibody array analysis also revealed alterations in multiple factors associated with inflammation and wound repair (**Supplementary Figures 5A, B**). ELISA results indicated that IL-2 and IL-6 levels decreased by day 7 and continued to decline by day 14, with the LZH+ZJ group showing the most pronounced reductions. IL-4 levels did not differ significantly from controls at either time point, whereas IL-10 increased on day 7 and rose further on day 14, with the LZH+ZJ group exhibiting the highest levels (**Supplementary Figures 5C, D**).

### 3.3 LZH, ZJ, and LZH+ZJ promote angiogenesis in HUVECs

**Figure 3:**
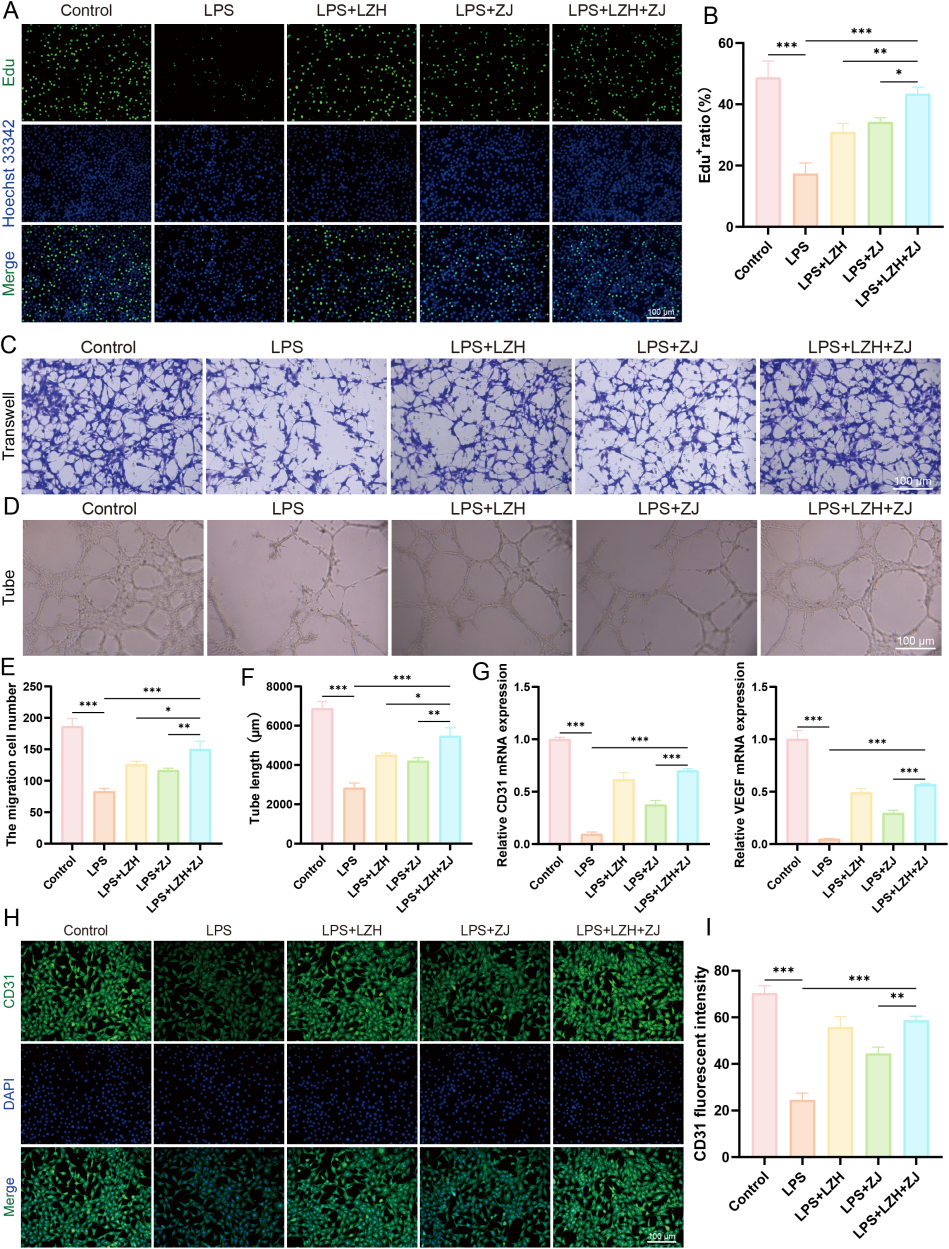
LZH, ZJ, and LZH+ZJ promote angiogenesis in HUVECs. **(A, B)** Representative images of EdU staining showing cell proliferation under different treatments and Quantification of EdU-positive cell ratio (%) in each group. Scale bar = 100 µm. (n = 3). **(C)** Representative images of Transwell migration assay for HUVEC cells under different treatments. Scale bar = 100 µm. **(D)** Representative images of tube formation assay under different treatments. Scale bar = 100 µm. **(E)** Quantification of migrated cells in the Transwell assay. (n = 3). **(F)** Quantification of total tube length formed by cells. (n = 3). **(G)** Relative mRNA expression levels of CD31 and VEGF measured by qRT-PCR in HUVEC cells under different treatments. (n = 3). **(H, I)** Immunofluorescence staining and quantitative analysis of CD31. Scale bar = 100 µm. (n = 3). Data is presented as mean ± SD. *P*-values are calculated using one-way analysis of variance (ANOVA) for multiple group comparisons. ^*^P<0.05, ^**^P<0.01, ^***^P<0.001.

To explore the pro-angiogenic effects of LZH, ZJ and LZH+ZJ, we first optimized the treatment concentrations using CCK-8 assays. Based on these results, 0.125% LZH and 0.25% ZJ were selected for subsequent experiments (**Supplementary Figures 6A, B**). In addition, LPS treatment reduced HUVEC viability in a dose-dependent manner, and 100 μg/mL LPS was selected to establish the *in vitro* inflammatory model (**Supplementary Figure 6C**). Notably, a 1:1 ratio mixture of LZH and ZJ (50% LZH + 50% ZJ) resulted in the highest cell viability and was therefore used for LZH+ZJ treatments in the following studies (**Supplementary Figure 6D**). EdU assay results demonstrated that combined treatment with LZH and ZJ significantly enhanced HUVEC proliferation and was superior to monotherapy (**Figures 3A, B**). Consistently, Transwell assays demonstrated a marked increase in HUVEC migration in the LZH+ZJ group (**Figures 3C, E**). Furthermore, tube formation assays revealed that the angiogenic capacity of HUVECs was significantly enhanced by LZH+ZJ relative to the single-agent groups (**Figures 3D, F**). RT-PCR analysis demonstrated that CD31 and VEGF mRNA expression levels were significantly upregulated in the combination group compared with LPS group (**Figure 3G**). Western blot results were consistent with the RT-PCR findings (**Supplementary Figure 7A**). Immunofluorescence staining further demonstrated increased expression of CD31 and VEGF in HUVECs treated with LZH+ZJ (**Figures 3H, I**; **Supplementary Figures 7B, C**).

### 3.4 Anti-inflammatory effects of LZH, ZJ, and LZH+ZJ

**Figure 4:**
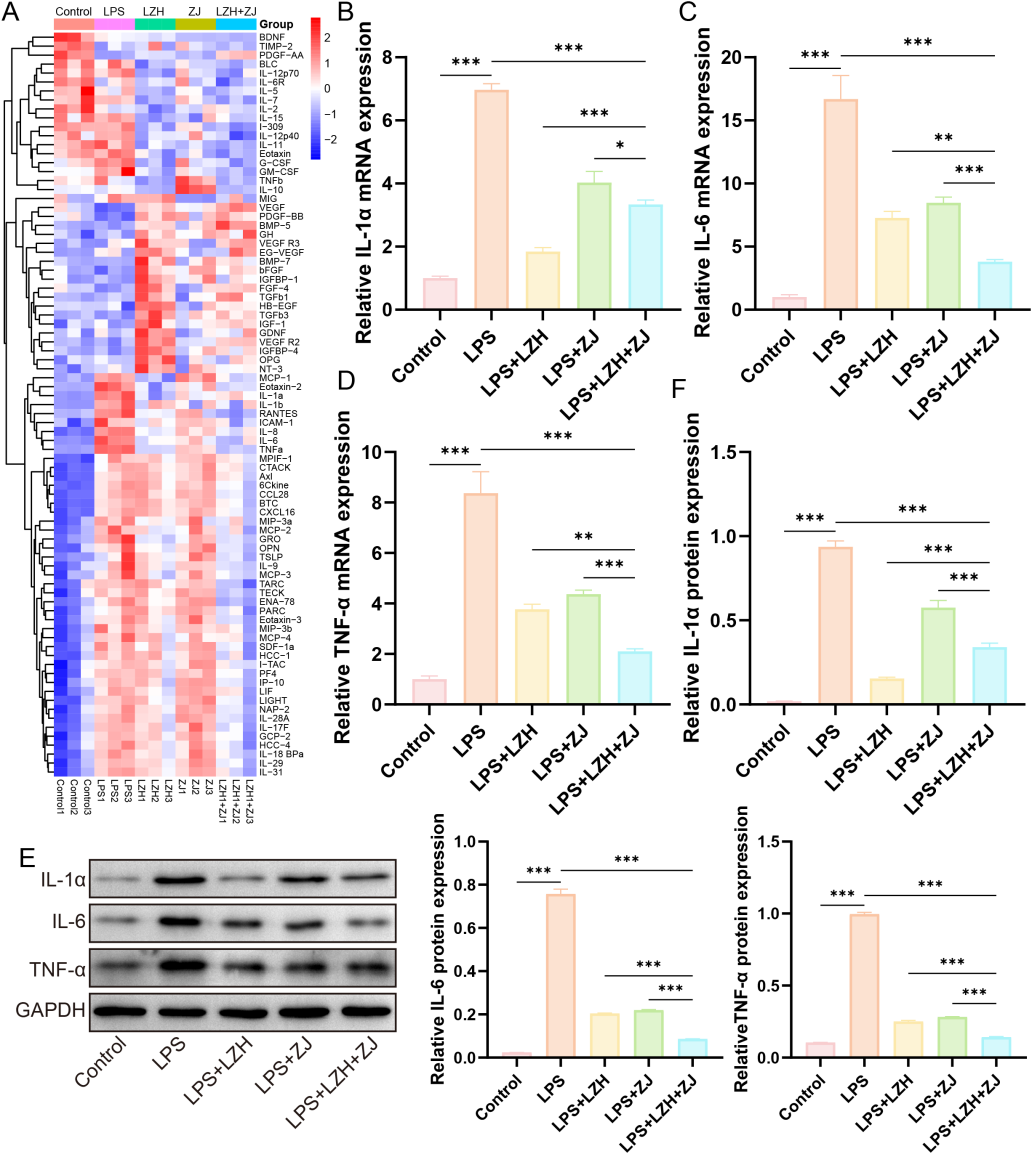
Anti-inflammatory effects of LZH, ZJ, and LZH+ZJ. **(A)** Heatmap showing the differential expression of multiple inflammatory, cytokines and growth factors across different treatment groups. **(B-D)** Relative mRNA expression levels of IL-1α, IL-6, and TNF-α in each group measured by quantitative RT-PCR. (n = 3). **(E, F)** Western blot analysis of IL-1α, IL-6, and TNF-α protein expression in different treatment groups. (n = 3). Data is presented as mean ± SD. *P*-values are calculated using one-way analysis of variance (ANOVA) for multiple group comparisons. ^*^P<0.05, ^**^P<0.01, ^***^P<0.001.

Cytokine array analysis showed that treatment with LZH, ZJ, and LZH+ZJ significantly reduced the levels of multiple inflammatory mediators in the cell culture supernatants (**Figure 4A**). RT-PCR and Western blot analyses demonstrated that LPS stimulation markedly increased the expression of TNF-α, IL-6, and IL-1α compared to the control group, while treatment with LZH, ZJ, or LZH+ZJ significantly suppressed the expression of these inflammatory cytokines. Among them, LZH+ZJ exhibited the most pronounced anti-inflammatory effect (**Figures 4B–F**).

### 3.5 Network pharmacology-based analysis

**Figure 5:**
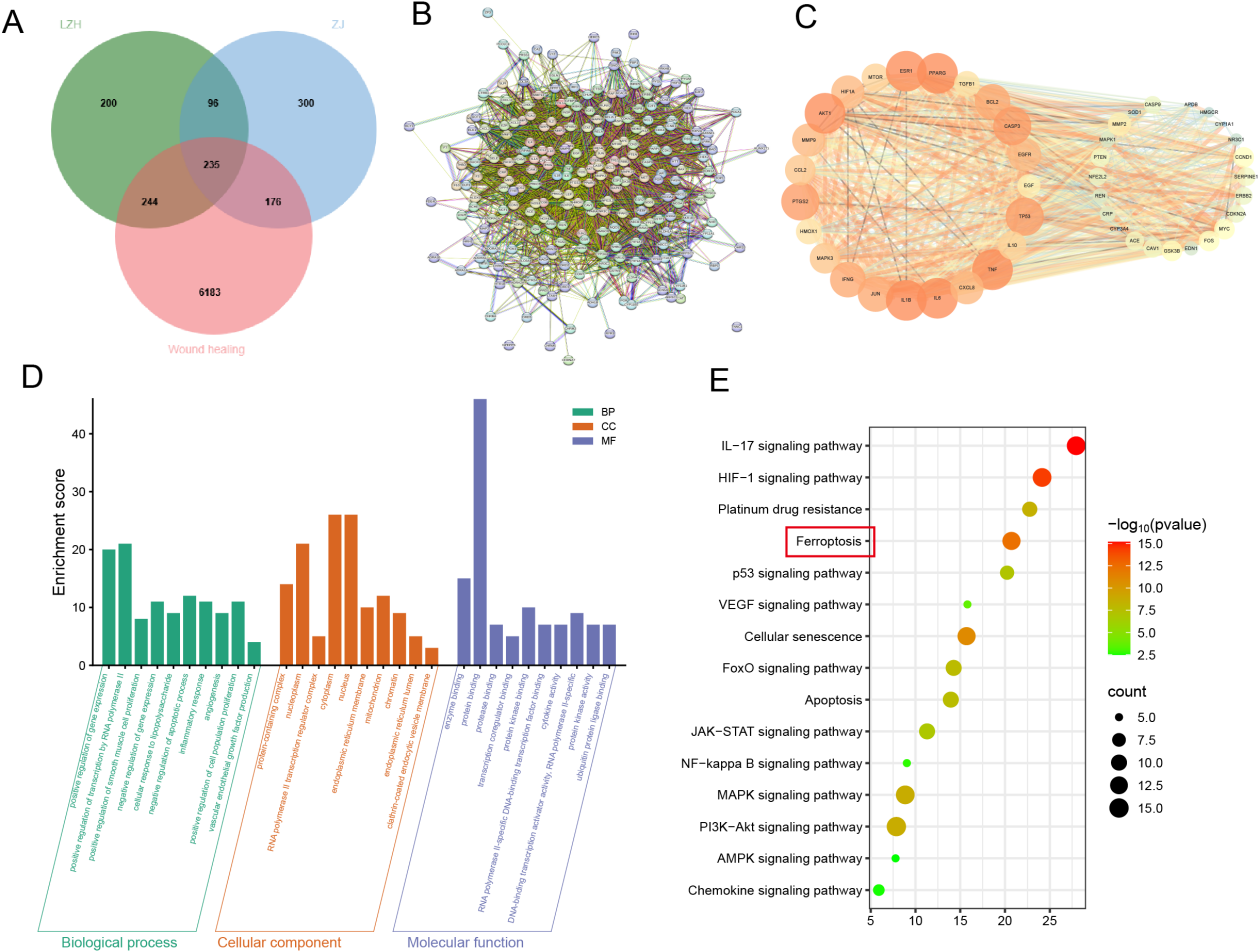
Network pharmacology-based analysis. **(A)** Venn diagrams showing the targets of LZH-ZJ-wound healing. **(B)** PPI networks of LZH-ZJ-wound healing. **(C)** Core target maps of LZH-ZJ-wound healing. **(D, E)** GO and KEGG enrichment analyses results for LZH-ZJ-wound healing.

To elucidate the molecular mechanisms by which LZH and ZJ combination therapy promotes wound healing, we performed network pharmacology analysis. Venn diagram analysis revealed the intersection between predicted drug targets and disease-related genes, identifying 479 LZH-related and 411 ZJ-related targets associated with wound healing (**Supplementary Figures 8A, B**). Additionally, 236 common targets were shared among LZH, ZJ, and wound healing (**Figure 5A**). Based on the compounds and core targets, drug-component-target networks for LZH and ZJ were constructed and visualized using Cytoscape, with the LZH network comprising 689 nodes and 3,013 edges (**Supplementary Figure 8C**), and the ZJ network consisting of 502 nodes and 1,317 edges (**Supplementary Figure 8D**). To identify core targets of LZH, ZJ, and LZH+ZJ, protein-protein interaction (PPI) networks for LZH-wound healing, ZJ-wound healing, and LZH-ZJ-wound healing were analyzed using the STRING database (**Figure 5B**; **Supplementary Figures 8E, F**). The PPI data were imported into Cytoscape for visualization and further analysis, which identified 95 core targets associated with wound healing for LZH, 91 for ZJ, and 46 for the combined LZH-ZJ treatment (**Figure 5C**; **Supplementary Figures 8G, H**).

To further explore the biological interactions and differential mechanisms of active compounds in LZH, ZJ, and their combination during wound healing, Gene Ontology (GO) and Kyoto Encyclopedia of Genes and Genomes (KEGG) pathway enrichment analyses were conducted. For LZH, GO enrichment analysis indicated that the predicted targets were mainly involved in biological processes related to inflammatory response, oxidative stress, and cell proliferation. Consistently, KEGG pathway analysis showed significant enrichment in the NF-κB signaling pathway, MAPK signaling pathway, and infection-related pathways (**Supplementary Figures 8I, J**). For ZJ, GO analysis demonstrated enrichment in biological processes associated with immune regulation, response to stimulus and cellular antioxidant responses. KEGG analysis further revealed that the targets were predominantly enriched in cytokine signaling, immune response, and VEGF signaling pathways and Toll-like receptor signaling pathway (**Supplementary Figures 8K, L****).** In contrast, the LZH+ZJ combination group exhibited a broader and more complex enrichment profile. GO analysis showed significant enrichment in angiogenesis, oxidative stress response, and inflammatory response. KEGG pathway analysis identified enrichment in multiple signaling pathways, including PI3K-Akt, VEGF, MAPK, NF-κB, and ferroptosis (**Figures 5D, E**). Notably, pathways such as ferroptosis and VEGF signaling were more prominently enriched in the combination group compared with either monotherapy. Overall, although several GO terms and KEGG pathways were shared among the three groups, distinct enrichment patterns were observed. These results collectively suggest that ferroptosis, inflammation, and oxidative stress represent key biological processes through which the bioactive components of LZH and ZJ contribute to wound healing.

### 3.6 LZH, ZJ, and LZH+ZJ suppress ferroptosis in *in vitro*

**Figure 6:**
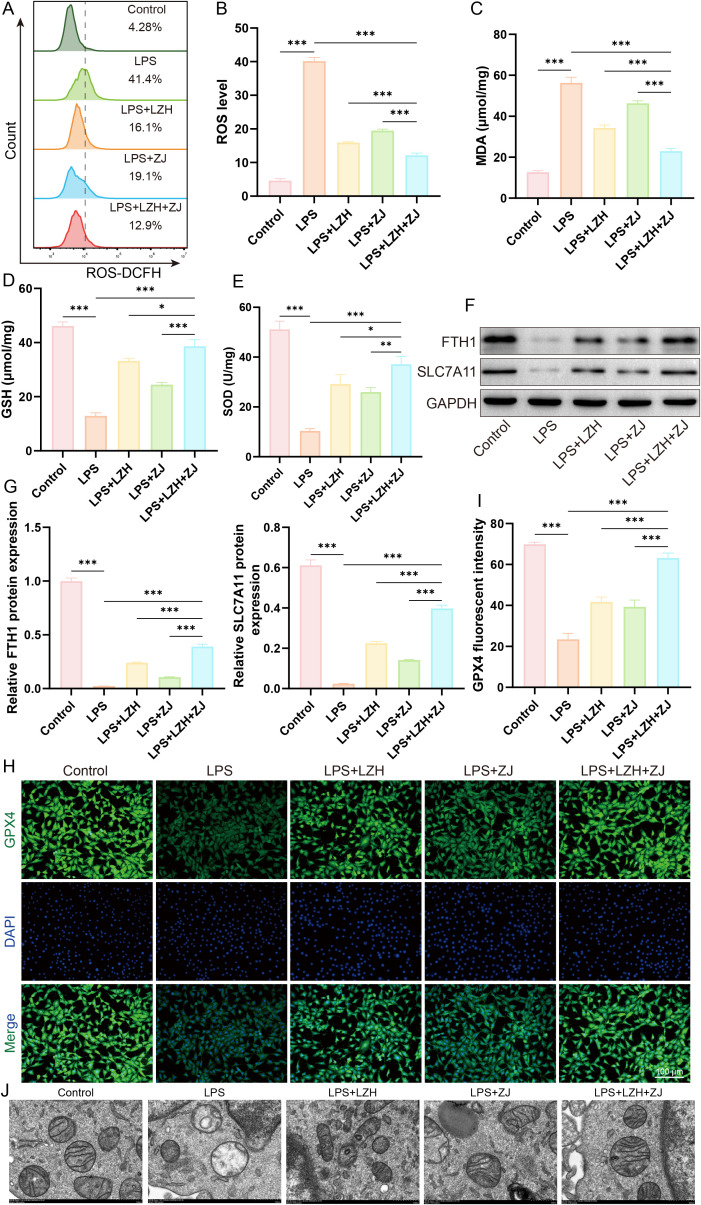
LZH, ZJ, and LZH+ZJ suppress ferroptosis in *in vitro*. **(A, B)** Flow cytometry measurement of intracellular ROS levels in HUVECs. (n = 3). **(C-E)** ELISA quantification of SOD, GSH, and MDA levels. (n = 3). **(F, G)** Western blot analysis and quantitative analysis of FTH1 and SLC7A11 proteins. (n = 3). **(H, I)** Immunofluorescence staining and quantitative analysis of GPX4. Scale bar = 100 µm. (n = 3). **(J)** Transmission electron microscopy was used to examine mitochondrial ultrastructure. (n = 3). Data is presented as mean ± SD. *P*-values are calculated using one-way analysis of variance (ANOVA) for multiple group comparisons. ^*^P<0.05, ^**^P<0.01, ^***^P<0.001.

Given the predicted ferroptosis involvement, ferroptosis-related indices were evaluated. Flow cytometry revealed that intracellular ROS levels were markedly reduced in the LZH+ZJ group compared with either monotherapy or the control group (**Figures 6A, B**). ELISA analysis showed that the combined treatment significantly enhanced cellular antioxidant capacity, as reflected by increased SOD and GSH levels accompanied by a pronounced decrease in MDA levels (**Figures 6C–E**). Consistently, Western blotting and immunofluorescence staining revealed that ferroptosis-associated proteins SLC7A11, FTH1 and GPX4 markedly upregulated in the LZH+ZJ group relative to either single-agent treatment (**Figures 6F–I**). Moreover, TEM revealed that mitochondrial integrity was better preserved following LZH+ZJ administration than with either individual agent (**Figure 6J**).

### 3.7 LZH and ZJ combination inhibits ferroptosis via activation of BMP5

**Figure 7:**
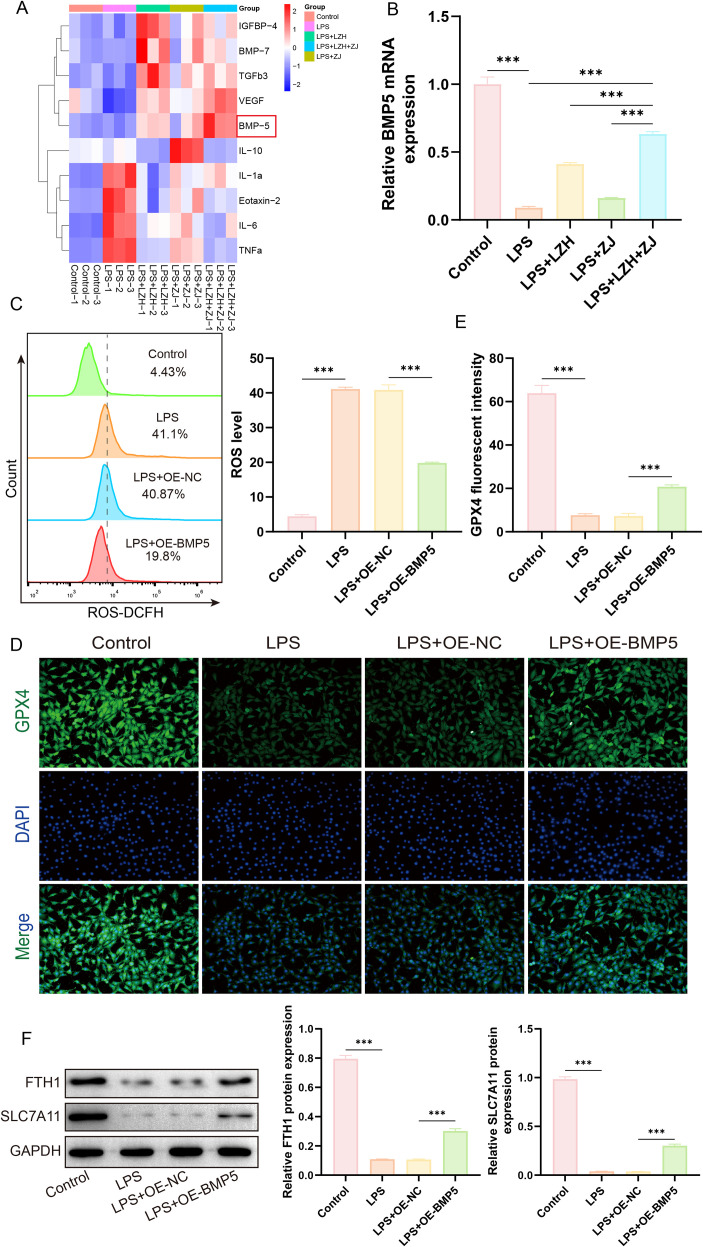
LZH and ZJ combination inhibits ferroptosis via activation of BMP5. **(A)** Heatmap showing the expression of differential molecules in the protein chip. **(B)** Relative mRNA expression levels of BMP5 measured by qRT-PCR under each treatment condition. (n = 3). **(C)** Flow cytometry measurement of intracellular ROS levels in HUVECs. (n = 3). **(D, E)** Immunofluorescence staining and quantitative analysis of GPX4. Scale bar = 100 µm. (n = 3). **(F)** Western blot analysis and quantitative analysis of FTH1 and SLC7A11 proteins. (n = 3). Data is presented as mean ± SD. *P*-values are calculated using one-way analysis of variance (ANOVA) for multiple group comparisons. ^*^P<0.05, ^**^P<0.01, ^***^P<0.001.

**Figure 8:**
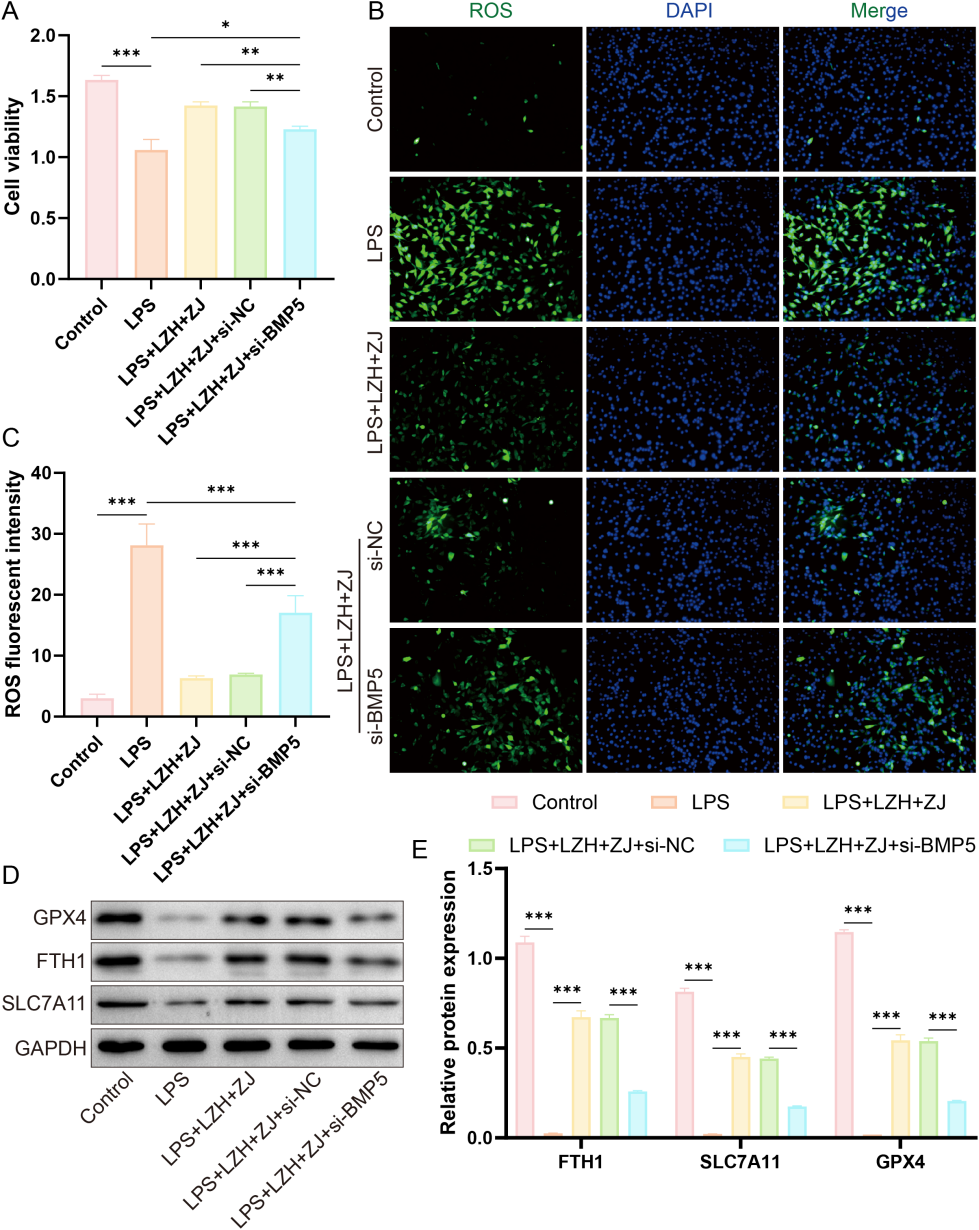
**(A)** Cell viability following BMP5 knockdown was assessed using the CCK-8 assay. (n = 3). **(B, C)** Intracellular ROS levels after BMP5 knockdown were evaluated by immunofluorescence staining. (n = 3). **(D, E)** Protein expression levels of GPX4, FTH1, and SLC7A11 after BMP5 knockdown were determined by Western blot analysis. (n = 3). Data is presented as mean ± SD. *P*-values are calculated using one-way analysis of variance (ANOVA) or two-way analysis of variance (ANOVA) for multiple group comparisons. ^*^P<0.05, ^**^P<0.01, ^***^P<0.001.

Proteomic array analysis revealed multiple differentially expressed proteins among the treatment groups, with BMP5 significantly upregulated in the LZH+ZJ combination group (**Figure 7A**). RT-PCR analysis confirmed that BMP5 expression was significantly elevated following LZH+ZJ treatment (**Figure 7B**). To further elucidate the underlying molecular mechanisms, we investigated the role of BMP5 in regulating ferroptosis. Co-IP assays indicated that BMP5 may interact with GPX4, suggesting a potential regulatory link between BMP5 and GPX4 in ferroptosis, whereas no detectable interaction was observed with SLC7A11 or FTH1 (**Supplementary Figure 9**). Next, we established a BMP5-overexpressing HUVEC model. Flow cytometry demonstrated a marked reduction in intracellular ROS accumulation in the LPS+OE-BMP5 group compared with LPS and LPS+OE-NC groups (**Figure 7C**). ELISA assays showed that BMP5 overexpression led to significantly increased levels of SOD and GSH, accompanied by a notable decrease in MDA levels (**Supplementary Figures 10A–C**). Immunofluorescence and western blot analyses further indicated that BMP5 overexpression markedly upregulated the ferroptosis-associated proteins GPX4, SLC7A11, and FTH1 (**Figures 7D–F**). To verify the role of BMP5, rescue experiments were performed. The results showed that, compared with the LPS group, treatment with LZH+ZJ significantly enhanced cell proliferation, reduced ROS levels, and upregulated GPX4, SLC7A11, and FTH1 expression, whereas BMP5 knockdown reversed these effects (**Figures 8A–E**).

## 4 Discussion

Skin wound healing is a dynamic, highly coordinated process involving multiple biological processes: inflammation, angiogenesis, extracellular matrix remodeling, and re-epithelialization. Under pathological conditions, disruptions to these processes impair normal repair, leading to delayed healing or chronic non-healing wounds [1, 32, 33]. In this study, we systematically evaluated the therapeutic potential of two TCM formulations, LZH and ZJ, administered individually or in combination, in skin wound healing. Using an integrated approach encompassing liquid chromatography-tandem mass spectrometry, *in vivo* and *in vitro* functional assays, network pharmacology, protein array profiling, and mechanistic validation, we demonstrated that LZH and ZJ, either alone or in combination, promote angiogenesis, attenuate inflammatory responses, and inhibit ferroptosis, thereby facilitating wound repair. Importantly, BMP5 was identified as a central regulatory node linking these biological processes, with BMP5-mediated regulation of ferroptosis playing a critical role in enhancing the healing response. Collectively, these findings elucidate the mechanistic basis underlying the synergistic effects of LZH and ZJ in promoting wound repair and highlight the therapeutic potential of multi-component herbal formulations for treating pathological wounds.

LC-MS/MS analysis confirmed that both LZH and ZJ are rich in polysaccharides, triterpenoids, flavonoids, and antioxidant compounds derived from *Ganoderma lucidum*, *Lentinula edodes*, *Tremella fuciformis*, *Lycium barbarum*, and other medicinal herbs. A growing body of evidence indicates that polysaccharides derived from these species exhibit pronounced pro-healing effects by modulating macrophage activation, reducing ROS accumulation, and enhancing tissue regeneration [34–36]. For instance, polysaccharides from *Ganoderma lucidum* have been shown to accelerate wound healing in diabetic models by regulating macrophage polarization and alleviating oxidative stress [25]. Similarly, Zhong et al. [37] reported that *Lycium barbarum* polysaccharides promote bone tissue repair through ROS scavenging and enhanced regenerative capacity, suggesting potential relevance to skin and soft tissue wound healing. Mapoung et al. [38] further demonstrated that polysaccharides extracted from *Auricularia auricula-judae* stimulate fibroblast and keratinocyte proliferation and migration, increase collagen synthesis, and significantly accelerate wound closure in a murine skin wound model. Mineroff et al. [18] further highlighted the therapeutic potential of Tremella fuciformis polysaccharides in skin protection, emphasizing their antioxidant, anti-inflammatory, and wound-healing properties. Consistent with these findings, our *in vivo* data show that both LZH and ZJ markedly enhance wound contraction, increase collagen deposition, and upregulate angiogenic markers, including CD31 and VEGF. Notably, combined treatment exhibited superior therapeutic efficacy to monotherapy, indicating complementary or synergistic interactions between their bioactive components.

Persistent inflammation and excessive oxidative stress are widely recognized as critical impediments to effective wound healing [4, 39]. In the present study, combined treatment with LZH and ZJ markedly reduced intracellular ROS levels and enhanced antioxidant capacity, as evidenced by increased SOD and GSH levels alongside decreased MDA accumulation. Concomitantly, this combination therapy significantly suppressed the expression of key pro-inflammatory cytokines (TNF-α, IL-6, and IL-1α) in both HUVECs and wound tissues. These findings align with previous studies showing that polysaccharide-rich herbal formulations can restore immune homeostasis and attenuate oxidative stress during tissue repair. For instance, Zhen et al. [40] reported that *Astragalus* polysaccharides promote diabetic wound healing by inducing macrophage polarization toward the anti-inflammatory M2 phenotype, thereby suppressing excessive inflammation response. Similarly, Zhao et al. [41] found that *Bletilla striata* polysaccharides inhibit NLRP3 inflammasome activation, reduce IL-1α and TNF-α expression, decrease ROS accumulation, and enhance angiogenesis and tissue regeneration. Notably, cytokine profiling revealed distinct immunoregulatory patterns among the treatment groups. IL-10 expression was markedly higher in the ZJ monotherapy group compared with both LZH and LZH + ZJ groups. As a key anti-inflammatory cytokine, IL-10 plays a central role in suppressing excessive immune responses and promoting the resolution of inflammation. The elevated IL-10 levels observed in the ZJ group may be attributed to its polysaccharide-rich composition derived from ingredients such as Lentinula edodes and Tremella fuciformis, which have been widely reported to possess potent immunomodulatory and anti-inflammatory properties. Roszczyk et al. demonstrated that polysaccharides from Lentinula edodes can exert immunoregulatory and anti-inflammatory effects by modulating cytokines such as IL-10, whereas Chen et al. reported that Tremella fuciformis polysaccharides can increase serum IL-10 levels in mice [21, 42]. Interestingly, although the combined LZH + ZJ treatment also increased IL-10 levels relative to the control group, its expression was lower than that observed with ZJ alone. This phenomenon may suggest a more balanced immunoregulatory effect induced by the combination therapy. Mechanistically, while ZJ appears to preferentially enhance anti-inflammatory signaling, LZH may be more closely associated with pathways involved in immune activation and inflammatory modulation. When used in combination, these two formulations may interact to achieve a more coordinated regulation of both pro- and anti-inflammatory pathways, rather than excessively amplifying a single anti-inflammatory mediator such as IL-10. Importantly, this differential cytokine pattern is consistent with the results of our network pharmacology analysis. As shown in the comparative enrichment analysis, ZJ targets were more prominently enriched in immune-regulatory and cytokine-related pathways, whereas LZH targets were more associated with inflammatory signaling pathways such as NF-κB and MAPK. In contrast, the combined LZH + ZJ treatment exhibited a broader and more integrated enrichment profile, involving both pro- and anti-inflammatory pathways, as well as oxidative stress-, angiogenesis-, and ferroptosis-related signaling. From a clinical perspective, this differential regulation of IL-10 may have important implications. Excessive IL-10 expression, although beneficial for suppressing inflammation, may potentially impair early immune responses required for effective wound debridement and host defense. Therefore, the relatively moderated IL-10 level observed in the combination group may be advantageous for maintaining an optimal inflammatory balance, thereby facilitating efficient wound healing. Consistent with these findings, the attenuation of inflammation and oxidative stress in the LZH + ZJ group likely underpins the enhanced angiogenesis and extracellular matrix remodeling observed during the proliferative and remodeling phases of wound healing. By alleviating chronic inflammation and oxidative damage, combined LZH + ZJ treatment restores a wound microenvironment conducive to cellular proliferation, migration, and matrix deposition. This restoration of tissue homeostasis not only accelerates regeneration but may also reduce the risk of chronic wound formation. Collectively, these results demonstrate that both individual and combined administration of LZH and ZJ exert therapeutic effects by coordinately regulating inflammatory responses and oxidative stress, thereby promoting efficient and sustained wound healing.

From the perspective of TCM, wound healing is governed by the principle of “treating the same disease with different therapeutic strategies,” in which multiple pathological processes such as inflammation, oxidative stress, and tissue regeneration are targeted through distinct yet complementary approaches. LZH is primarily considered to exert its therapeutic effects by reinforcing vital energy and modulating immune function, thereby alleviating excessive inflammatory responses. In contrast, ZJ is characterized by its ability to restore physiological balance, particularly through antioxidant properties that mitigate oxidative stress and maintain redox homeostasis. In our animal experiments, LZH demonstrated greater efficacy in reducing inflammatory cytokine levels in wound model mice, highlighting its anti-inflammatory activity. Conversely, ZJ exhibited more pronounced antioxidant effects, suggesting a distinct role in scavenging reactive oxygen species and protecting tissues from oxidative damage. Notably, combined treatment with LZH and ZJ showed greater therapeutic efficacy than either formulation alone, indicating potential synergistic effects. These findings suggest that a combinatorial therapeutic strategy targeting multiple aspects of wound healing may lead to improved outcomes. This observation further supports the concept that multi-component interventions can achieve enhanced efficacy in complex pathological conditions such as wound repair.

Beyond inflammation and angiogenesis, regulated cell death pathways—particularly ferroptosis—are increasingly recognized as key drivers of chronic non-healing wounds [43, 44]. Notably, although ferroptosis has been extensively studied in diabetic wound models, recent evidence indicates that it can also be activated in non-diabetic contexts under conditions of excessive oxidative stress and inflammation [45]. As an iron-dependent form of regulated cell death driven by lipid peroxidation and ROS accumulation, ferroptosis disrupts the function and viability of key cells involved in tissue repair, thereby impairing re-epithelialization, extracellular matrix formation, and angiogenesis [46]. In contrast to diabetic wounds, where ferroptosis is largely driven by hyperglycemia-induced oxidative stress, its activation in non-diabetic wounds is more closely associated with local inflammatory dysregulation and depletion of antioxidant defenses, ultimately delaying the normal progression of wound healing [44]. In the present study, network pharmacology analysis revealed that the therapeutic targets of combined LZH and ZJ treatment were significantly enriched in oxidative stress– and ferroptosis-related pathways, which was supported by our experimental findings. Compared with either formulation alone, combined LZH and ZJ treatment more effectively suppressed ROS accumulation, enhanced antioxidant defense-as indicated by increased SOD and GSH levels- decreased lipid peroxidation, as reflected by decreased MDA levels, and markedly upregulated key ferroptosis-associated proteins including GPX4, SLC7A11, and FTH1. Furthermore, transmission electron microscopy revealed preservation of mitochondrial ultrastructure following combination therapy, providing additional ultrastructural evidence of ferroptosis inhibition. These observations are consistent with recent reports demonstrating the pathological role of ferroptosis in impaired wound healing. For example, Wang et al. [47] showed that pharmacological inhibition of ferroptosis using Ferrostatin-1 significantly attenuates ferroptosis-associated inflammation and improves diabetic wound healing. Similarly, Jin et al. [48] reported that reducing iron overload and suppressing lipid peroxidation enhances endothelial cell function and promotes wound repair in a diabetic mouse model. Collectively, these data suggest that combined LZH and ZJ treatment mitigates chronic wound deterioration by inhibiting lipid peroxidation and restoring redox homeostasis, thereby representing a promising therapeutic strategy for refractory wound healing.

Protein array analysis revealed that BMP5 was significantly upregulated following combined treatment with LZH and ZJ, a finding further validated by RT-PCR. Members of the BMP family are well-recognized regulators of oxidative stress, inflammation, and tissue regeneration [49]. Although direct studies on BMP5 remain limited, mechanistic insights can be inferred from related BMP family members. For instance, BMP2 and BMP6 play key roles in regulating hepcidin expression and maintaining systemic iron homeostasis, with BMP–SMAD signaling implicated in iron-related disorders [50, 51]. Bone morphogenetic protein 7 (BMP7) has been shown to enhance diabetic wound healing by attenuating inflammation and promoting macrophage polarization toward the anti-inflammatory M2 phenotype [52]. Notably, recent studies reported that BMP7 inhibits ferroptosis in the kidney and pancreas of diabetic mice by suppressing lipid peroxidation, restoring GSH levels, and alleviating tissue injury [53]. In addition, Liang et al. [54] reported that BMP7 promotes stem cell migration and angiogenesis, highlighting its role in tissue regeneration and neovascularization. Consistent with these findings, our data show that combined LZH and ZJ treatment significantly upregulated BMP5 mRNA expression. Importantly, functional assays demonstrated that BMP5 overexpression in HUVECs reproduced the anti-ferroptotic effects of the combined treatment, including reduced ROS accumulation, enhanced antioxidant capacity, and increased expression of key ferroptosis-related proteins. Conversely, BMP5 knockdown markedly weakened the protective effects of LZH + ZJ co-treatment. Collectively, these findings suggest that BMP5 is a key functional mediator of the synergistic anti-ferroptotic effects of LZH and ZJ and may represent an important mechanistic axis through which the combined therapy promotes wound healing.

In addition to its direct role in regulating ferroptosis, BMP5 may also influence wound healing through modulation of other key biological processes, such as inflammation and angiogenesis. Members of the BMP family have been widely reported to participate in immune regulation and vascular remodeling. For example, BMP signaling has been shown to suppress excessive inflammatory responses by modulating cytokine production and macrophage polarization, thereby creating a favorable microenvironment for tissue repair. Moreover, BMP-related pathways have been implicated in endothelial cell proliferation, migration, and neovascularization, which are essential for effective wound healing. Given that our experimental data demonstrate that LZH + ZJ treatment significantly reduces pro-inflammatory cytokines (TNF-α, IL-6, IL-1α) and enhances angiogenic markers (CD31, VEGF), it is plausible that BMP5 may act as an upstream regulator coordinating these processes. Therefore, the beneficial effects of BMP5 activation on wound healing are likely not limited to ferroptosis inhibition, but may also involve indirect regulation of inflammation and angiogenesis. However, further studies are required to determine whether these effects are directly mediated by BMP5 or occur through downstream signaling crosstalk.

Although this study provides important insights into the role of BMP5 in wound healing, several limitations should be considered. First, the *in vitro* experiments were conducted solely in HUVECs, which capture only one aspect of the multicellular and highly dynamic wound microenvironment. Given that wound repair is orchestrated by coordinated interactions among keratinocytes, fibroblasts, endothelial cells, and immune cells, future studies incorporating multiple relevant cell types will be essential to more comprehensively delineate the cellular specificity and intercellular crosstalk underlying the therapeutic effects of LZH + ZJ. Second, although consistent pro-healing effects were observed in both *in vivo* and *in vitro* models, validation in human-derived tissues or clinical samples is still lacking. The inclusion of patient-derived specimens or clinically relevant models would therefore strengthen the translational significance and clinical applicability of our findings. Third, while BMP5 was identified as a critical mediator associated with ferroptosis inhibition and enhanced wound healing, its causal role *in vivo* has not yet been definitively established. Future studies employing gain- and loss-of-function strategies targeting BMP5 in animal models will be necessary to confirm its functional importance and to exclude potential compensatory effects within the BMP signaling network. Finally, although this study delineates a BMP5-centered anti-ferroptotic mechanism, the broader regulatory landscape underlying the multi-component and multi-target effects of LZH + ZJ remains incompletely understood. In particular, transcriptomic approaches such as bulk RNA sequencing were not performed, which may limit the identification of upstream regulators of BMP5 and additional signaling pathways beyond ferroptosis. Future integration of transcriptomic profiling will therefore be valuable for systematically elucidating the global molecular networks involved and for better capturing the holistic pharmacological characteristics of traditional Chinese medicine.

## 5 Conclusion

**Figure 9:**
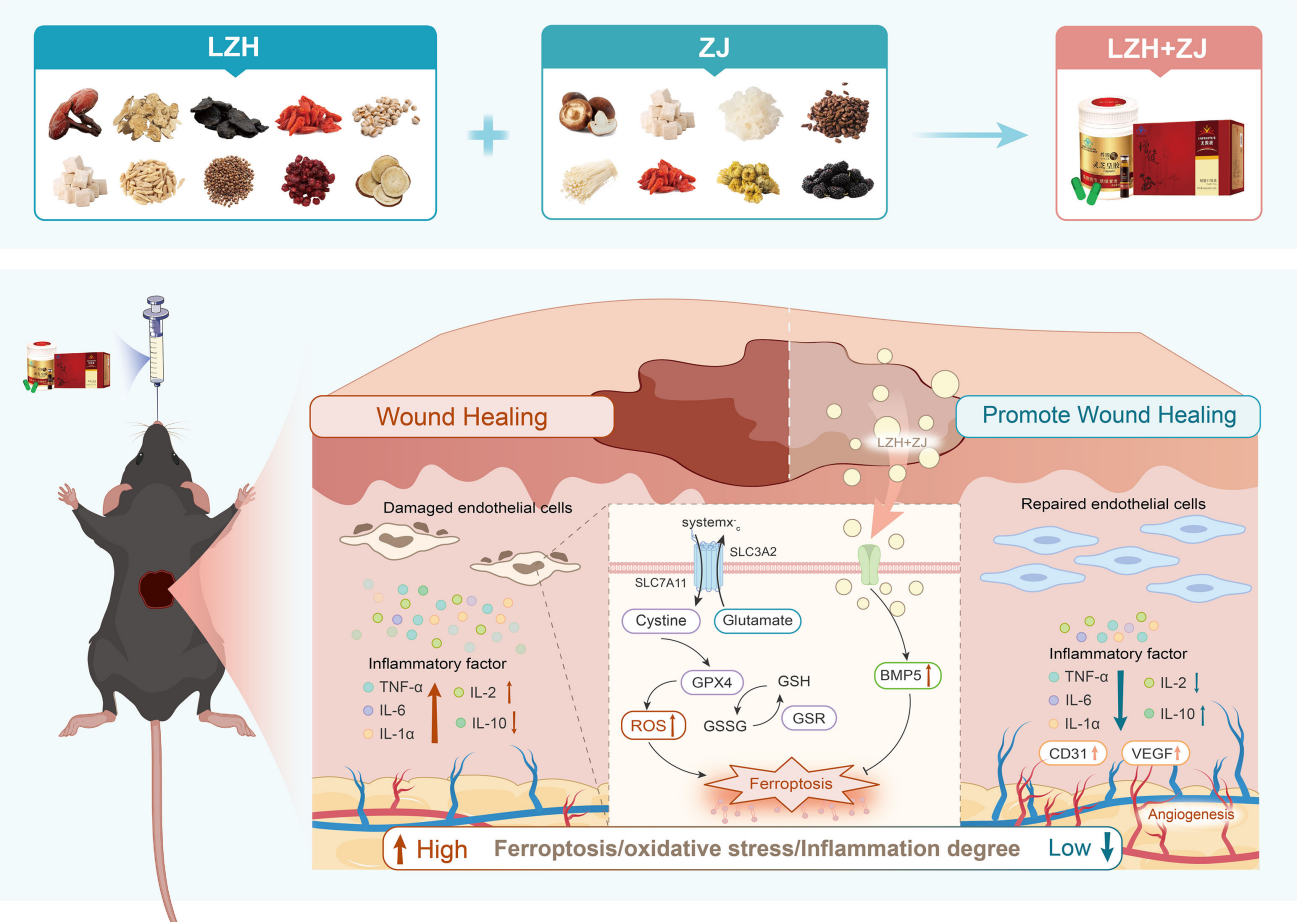
LZH+ZJ promotes wound healing by activating BMP5 to suppress ferroptosis, reduce oxidative stress, and suppress inflammatory responses.

In summary, this study demonstrates that Lingzhi Huang capsules and Zeng Jian Health Tonic, administered individually or in combination, significantly promote skin wound healing by enhancing angiogenesis, suppressing inflammatory responses, and alleviating oxidative stress. Importantly, the combined treatment exhibited superior efficacy and was shown to inhibit ferroptosis through activation of BMP5, identifying a previously unrecognized regulatory pathway linking traditional herbal medicine to redox-dependent cell death in wound repair (**Figure 9**). By integrating chemical profiling, functional assays, network pharmacology, and mechanistic validation, this work provides a comprehensive framework for understanding the multi-target therapeutic actions of complex herbal formulations. These findings not only offer mechanistic support for the traditional use of LZH and ZJ but also highlight BMP5-mediated ferroptosis inhibition as a promising strategy for improving wound healing outcomes.

## Data Availability

The original contributions presented in the study are included in the article/**Supplementary Material**. Further inquiries can be directed to the corresponding authors.

## References

[1] Peña OA Martin P . Cellular and molecular mechanisms of skin wound healing. Nat Rev Mol Cell Biol. (2024) 25:599–616. doi: 10.1038/s41580-024-00715-1. PMID: 38528155

[2] Liu Z Bian X Luo L Björklund ÅK Li L Zhang L . Spatiotemporal single-cell roadmap of human skin wound healing. Cell Stem Cell. (2025) 32:479–98:e8. doi: 10.1016/j.jid.2024.10.551. PMID: 39729995

[3] Gushiken LFS Beserra FP Bastos JK Jackson CJ Pellizzon CH . Cutaneous wound healing: An update from physiopathology to current therapies. Life. (2021) 11:665. doi: 10.3390/life11070665. PMID: 34357037 PMC8307436

[4] Mamun AA Shao C Geng P Wang S Xiao J . Recent advances in molecular mechanisms of skin wound healing and its treatments. Front Immunol. (2024) 15:1395479. doi: 10.3389/fimmu.2024.1395479. PMID: 38835782 PMC11148235

[5] Dong Y Wang Z . ROS-scavenging materials for skin wound healing: advancements and applications. Front Bioeng. Biotechnol. (2023) 11:1304835. doi: 10.3389/fbioe.2023.1304835. PMID: 38149175 PMC10749972

[6] Joorabloo A Liu T . Recent advances in reactive oxygen species scavenging nanomaterials for wound healing. Explor (Beijing). (2024) 4:20230066. doi: 10.1002/exp.20230066. PMID: 38939866 PMC11189585

[7] Daré RG Lopes LB Petri-Fink A Rothen-Rutishauser B . Advancing preclinical research with reconstructed *in vitro* skin models mimicking non-healing wounds. Int J Pharm X. (2026) 11:100518. doi: 10.1016/j.ijpx.2026.100518. PMID: 41909167 PMC13022694

[8] Tian K Wu P Gao S Xu C Xu W Jia Z . A deferoxamine-loaded microneedle patch enhances healing of radiation-induced skin injury: Potential involvement of ferroptosis. ACS Appl Mater. Interfaces. (2025) 17:15035–49. doi: 10.1021/acsami.4c21589. PMID: 40025663

[9] Jin C Zhang DP Lin Z Lin YZ Shi YF Dong XY . Piezo1-mediated ferroptosis delays wound healing in aging mice by regulating the transcriptional activity of SLC7A11 through activating transcription factor 3. Res (Wash. D. C). (2025) 8:718. doi: 10.34133/research.0718. PMID: 40463502 PMC12133029

[10] Du W Wang Z Dong Y Hu H Zhou H He X . Electroacupuncture promotes skin wound repair by improving lipid metabolism and inhibiting ferroptosis. J Cell Mol Med. (2023) 27:2308–20. doi: 10.1111/jcmm.17811. PMID: 37307402 PMC10424292

[11] Hua H Tang J-Y Zhao J-N Wang T Zhang J-H Yu J-Y . From traditional medicine to modern medicine: The importance of TCM regulatory science (TCMRS) as an emerging discipline. Chin Med. (2025) 20:92. doi: 10.1186/s13020-025-01152-8. PMID: 40563101 PMC12199503

[12] Fan M Jin C Li D Deng Y Yao L Chen Y . Multi-level advances in databases related to systems pharmacology in traditional Chinese medicine: A 60-year review. Front Pharmacol. (2023) 14:1289901. doi: 10.3389/fphar.2023.1289901. PMID: 38035021 PMC10682728

[13] Chinonso AD Kayode AA Adondua MA Chinekwu UK . Biochemistry of traditional herbal compounds and their molecular targets. Pharmacogn. Rev. (2025) 19(37):83–90. doi: 10.5530/phrev.20252302. PMID: 41936619

[14] Liu X Guo C Yang W Wang W Diao N Cao M . Composite microneedles loaded with Astragalus membranaceus polysaccharide nanoparticles promote wound healing by curbing the ROS/NF-κB pathway to regulate macrophage polarization. Carbohydr. Polym. (2024) 345:122574. doi: 10.1016/j.carbpol.2024.122574. PMID: 39227108

[15] He X Liu L Gu F Huang R Liu L Nian Y . Exploration of the anti-inflammatory, analgesic, and wound healing activities of Bletilla striata polysaccharide. Int J Biol Macromol. (2024) 261:129874. doi: 10.1016/j.ijbiomac.2024.129874. PMID: 38307430

[16] Jiao C Yun H Liang H Lian X Li S Chen J . An active ingredient isolated from Ganoderma lucidum promotes burn wound healing via TRPV1/SMAD signaling. Aging (Albany. NY). (2022) 14:5376–89. doi: 10.18632/aging.204119. PMID: 35696640 PMC9320545

[17] Ghiulai R Roşca OJ Antal DS Mioc M Mioc A Racoviceanu R . Tetracyclic and pentacyclic triterpenes with high therapeutic efficiency in wound healing approaches. Molecules. (2020) 25(23):5557. doi: 10.3390/molecules25235557. PMID: 33256207 PMC7730621

[18] Mineroff J Jagdeo J . The potential cutaneous benefits of Tremella fuciformis. Arch Dermatol Res. (2023) 315:1883–6. doi: 10.1007/s00403-023-02550-4. PMID: 36757441

[19] Zhao B Zhang X Han W Cheng J Qin Y . Wound healing effect of an Astragalus membranaceus polysaccharide and its mechanism. Mol Med Rep. (2017) 15:4077–83. doi: 10.3892/mmr.2017.6488. PMID: 28440420 PMC5436241

[20] Du W Wang Z Han M Zheng Y Tao B Pan N . Astragalus polysaccharide-containing 3D-printed scaffold for traumatized skin repair and proteomic study. J Cell Mol Med. (2024) 28:e70023. doi: 10.1111/jcmm.70023. PMID: 39158533 PMC11331928

[21] Roszczyk A Turło J Zagożdżon R Kaleta B . Immunomodulatory properties of polysaccharides from Lentinula edodes. Int J Mol Sci. (2022) 23(16):8980. doi: 10.3390/ijms23168980. PMID: 36012249 PMC9409024

[22] Lei H Zhao J Li H Fan D . Paramylon hydrogel: A bioactive polysaccharides hydrogel that scavenges ROS and promotes angiogenesis for wound repair. Carbohydr. Polym. (2022) 289:119467. doi: 10.1016/j.carbpol.2022.119467. PMID: 35483864

[23] Yin S Xia F Zou W Jiang F Shen K Sun B . Ginsenoside Rg1 regulates astrocytes to promote angiogenesis in spinal cord injury via the JAK2/STAT3 signaling pathway. J Ethnopharmacol. (2024) 334:118531. doi: 10.1016/j.jep.2024.118531. PMID: 38971343

[24] Tie L Yang HQ An Y Liu SQ Han J Xu Y . Ganoderma lucidum polysaccharide accelerates refractory wound healing by inhibition of mitochondrial oxidative stress in type 1 diabetes. Cell Physiol Biochem. (2012) 29:583–94. doi: 10.1159/000338512. PMID: 22508065

[25] Li F Liu T Liu X Han C Li L Zhang Q . Ganoderma lucidum polysaccharide hydrogel accelerates diabetic wound healing by regulating macrophage polarization. Int J Biol Macromol. (2024) 260:129682. doi: 10.1016/j.ijbiomac.2024.129682. PMID: 38266851

[26] Zhou T Zhang C Wang X Lin J Yu J Liang Y . Research on traditional Chinese medicine as an effective drug for promoting wound healing. J Ethnopharmacol. (2024) 332:118358. doi: 10.1016/j.jep.2024.118358. PMID: 38763370

[27] Hu H Sheng Q Yang F Wu X Zhang Y Wu S . Enhanced skin wound healing through chemically modified messenger RNA encoding epidermal growth factor (EGF). Int Wound J. (2025) 22:e70143. doi: 10.1111/iwj.70143. PMID: 40320617 PMC12050261

[28] Kim YS Lew DH Tark KC Rah DK Hong JP . Effect of recombinant human epidermal growth factor against cutaneous scar formation in murine full-thickness wound healing. J Kor. Med Sci. (2010) 25:589–96. doi: 10.3346/jkms.2010.25.4.589. PMID: 20358003 PMC2844601

[29] Li R Fan X He Z Shen H Wu H Chen J . IL-1β-stimulated bone mesenchymal stem cell-derived exosomes promote cutaneous wound healing by inhibiting SIRT6/NLRP3 pathway. Int Immunopharmacol. (2025) 166:115566. doi: 10.1016/j.intimp.2025.115566. PMID: 40976046

[30] Peng P Fan S Liu P Huang X Xuan Z Liu A . Porcine decellularized splenic matrix hydrogel-based vesicle sustained-release system: A dual-regulatory biomaterial for accelerated wound healing via angiogenesis and immune remodeling. Mater. Today Bio. (2025) 35:102601. doi: 10.1016/j.mtbio.2025.102601. PMID: 41427061 PMC12712692

[31] Mao J Lin Z Pan Z Huang Y Luo Y Xie H . Angiogenesis in skin injury repair: Mechanisms, regulation, and therapeutic strategies. Arch Dermatol Res. (2025) 317:1–19. doi: 10.1007/s00403-025-04424-3. PMID: 30311153

[32] Sun D Chang Q Lu F . Immunomodulation in diabetic wounds healing: The intersection of macrophage reprogramming and immunotherapeutic hydrogels. J Tissue Eng. (2024) 15:20417314241265202. doi: 10.1177/20417314241265202. PMID: 39071896 PMC11283672

[33] Zhang R Tan SF Wang Y Wu J Zhang C . Hydrogels incorporating active compounds from traditional Chinese medicine for diabetic wound healing: Mechanistic pathways and bioengineering progress. Front Cell Dev Biol. (2025) 13:1666646. doi: 10.3389/fcell.2025.1666646. PMID: 40995560 PMC12454359

[34] Mapoung S Umsumarng S Semmarath W Arjsri P Thippraphan P Yodkeeree S . Skin wound-healing potential of polysaccharides from medicinal mushroom Auricularia auricula-judae (Bull.). J Fungi. (Basel). (2021) 7(4):247. doi: 10.3390/jof7040247. PMID: 33806146 PMC8064461

[35] Zhen Z Wei S Yunfei W Jie X Jienan X Yiting S . Astragalus polysaccharide improves diabetic ulcers by promoting M2-polarization of macrophages to reduce excessive inflammation via the β-catenin/ NF-κB axis at the late phase of wound-healing. Heliyon. (2024) 10:e24644. doi: 10.1016/j.heliyon.2024.e24644. PMID: 38390059 PMC10881534

[36] Zhao Y Wang Q Yan S Zhou J Huang L Zhu H . Bletilla striata polysaccharide promotes diabetic wound healing through inhibition of the NLRP3 inflammasome. Front Pharmacol. (2021) 12:659215. doi: 10.3389/fphar.2021.659215. PMID: 33981238 PMC8110216

[37] Zhao Y Chen Z Xie S Xiao F Hu Q Ju Z . The emerging role and therapeutical implications of ferroptosis in wound healing. Burns. Trauma. (2025) 13:tkae082. doi: 10.1093/burnst/tkae082. PMID: 39958433 PMC11827611

[38] Bi M Li D Zhang J . Research progress and insights on the role of ferroptosis in wound healing. Int Wound J. (2023) 20:2473–81. doi: 10.1111/iwj.14102. PMID: 36788729 PMC10333008

[39] Ru Q Li Y Chen L Wu Y Min J Wang F . Iron homeostasis and ferroptosis in human diseases: Mechanisms and therapeutic prospects. Signal Transd. Targ. Ther. (2024) 9:271. doi: 10.1038/s41392-024-01969-z. PMID: 39396974 PMC11486532

[40] Wang T Zheng Y Zhang J Wu Z . Targeting ferroptosis promotes diabetic wound healing via Nrf2 activation. Heliyon. (2024) 10:e37477. doi: 10.1016/j.heliyon.2024.e37477. PMID: 39421383 PMC11483302

[41] Jin C Lin YZ Zhang RY Zhang DP Xue KK Xu J . A multifunctional hydrogel promotes diabetic wound healing by remodeling iron balance and energy metabolism. Biomaterials. (2026) 326:123640. doi: 10.1016/j.biomaterials.2025.123640. PMID: 40848427

[42] Bansal P Kajal Sharma S Mazumder A . Role of BMP-7 in cardiovascular diseases: From molecular mechanisms to therapeutic horizons. Curr Drug Targets. (2025) 27(4):217–26. doi: 10.2174/0113894501421956251014101956. PMID: 41169133

[43] Zhang W He H Chen S Xie S Tong X Ding X . Research progress on the role and mechanisms of ferroptosis in diabetic wound repair. Cell Death Discov. (2025) 11:515. doi: 10.1038/s41420-025-02808-y. PMID: 41203620 PMC12594836

[44] Canali S Wang CY Zumbrennen-Bullough KB Bayer A Babitt JL . Bone morphogenetic protein 2 controls iron homeostasis in mice independent of Bmp6. Am J Hematol. (2017) 92:1204–13. doi: 10.1002/ajh.24888. PMID: 28815688 PMC5986189

[45] Xiao X Alfaro-Magallanes VM Babitt JL . Bone morphogenic proteins in iron homeostasis. Bone. (2020) 138:115495. doi: 10.1016/j.bone.2020.115495. PMID: 32585319 PMC7453787

[46] Da Silva J Figueiredo A Tseng Y-H Carvalho E Leal EC . Bone morphogenetic protein 7 improves wound healing in diabetes by decreasing inflammation and promoting M2 macrophage polarization. Int J Mol Sci. (2025) 26:2036. doi: 10.3390/ijms26052036. PMID: 40076659 PMC11900347

[47] Song SH Han D Park K Um JE Kim S Ku M . Bone morphogenetic protein-7 attenuates pancreatic damage under diabetic conditions and prevents progression to diabetic nephropathy via inhibition of ferroptosis. Front Endocrinol (Lausanne). (2023) 14:1172199. doi: 10.3389/fendo.2023.1172199. PMID: 37293506 PMC10244744

[48] Liu S Wang L Zhang Z Leng Y Yang Y Fu X . The potential of astragalus polysaccharide for treating diabetes and its action mechanism. Front Pharmacol. (2024) 15:1339406. doi: 10.3389/fphar.2024.1339406. PMID: 38659573 PMC11039829

[49] He J Zhou S Wang J Sun B Ni D Wu J . Anti-inflammatory and anti-oxidative electrospun nanofiber membrane promotes diabetic wound healing via macrophage modulation. J Nanobiotechnol. (2024) 22:116. doi: 10.1186/s12951-024-02385-9. PMID: 38493156 PMC10943854

[50] Li Y Cheng B Tian J . Platelet-rich plasma may accelerate diabetic wound healing by modulating epithelial/endothelial-mesenchymal transition through inhibiting reactive oxygen species-mediated oxidative stress. Front Bioeng. Biotechnol. (2025) 13:1623780. doi: 10.3389/fbioe.2025.1623780. PMID: 40861861 PMC12375923

[51] Li FL Wang GC Wu BQ . Clinical application of traditional Chinese medicine powder in the treatment of acute and chronic wounds. Int Wound J. (2023) 20:799–805. doi: 10.1111/iwj.13925. PMID: 36148625 PMC9927914

[52] Zhong W Liao W Xu L He N Xu K Liu C . Lycium-barbarum polysaccharide-loaded dual-crosslinked rigid hydrogel enhances bone healing in diabetic bone defects by scavenging reactive oxygen species. Adv Healthc. Mater. (2025) 14:e2404741. doi: 10.1002/adhm.202404741. PMID: 40095333 PMC12023829

[53] Chen L Chen J Li J Xie J Yu M Zhou M . Physicochemical properties and *in vitro* digestion behavior of a new bioactive Tremella fuciformis gum. Int J Biol Macromol. (2022) 207:611–21. doi: 10.1016/j.ijbiomac.2022.02.166. PMID: 35247431

[54] Liang C Liang Q Xu X Liu X Gao X Li M . Bone morphogenetic protein 7 mediates stem cells migration and angiogenesis: Therapeutic potential for endogenous pulp regeneration. Int J Oral Sci. (2022) 14:38. doi: 10.1038/s41368-022-00188-y. PMID: 35858911 PMC9300630

