# Supplementary material for: Combined Lingzhi Huang capsules and Zeng Jian health tonic accelerates skin wound healing via BMP5-mediated inhibition of ferroptosis
Source: Front Immunol. 2026 Jun 2;17:1818752. doi: 10.3389/fimmu.2026.1818752 (PMC13268888; doi:10.3389/fimmu.2026.1818752)
Supplement: Supplementary file 1 [file DataSheet1.docx]

**TableS1. LC-MS/MS data of characterized compounds in LZH**

| No. | Molecular weight (g/mol) | Formula | Compounds | Ion mode | Class |
| --- | --- | --- | --- | --- | --- |
|  | 182.079 | C6H14O6 | D-Sorbitol | M-H | Organooxygen compounds |
|  | 260.0297 | C6H13O9P | D-Fructose 1-phosphate | M-H | Organooxygen compounds |
|  | 215.0559 | C5H14NO6P | Glycerylphosphorylethanolamine | M-H | Glycerophospholipids |
|  | 342.1162 | C12H22O11 | Sucrose | M-H | Organooxygen compounds |
|  | 180.0634 | C6H12O6 | Fructose | M-H | Organooxygen compounds |
|  | 88.016 | C3H4O3 | Pyruvic acid | M-H | Keto acids and derivatives |
|  | 192.027 | C6H8O7 | Isocitrate | M-H | Carboxylic acids and derivatives |
|  | 134.0215 | C4H6O5 | L-Malic acid | M-H | Hydroxy acids and derivatives |
|  | 260.1372 | C11H20N2O5 | gamma-Glutamylisoleucine | M-H | Carboxylic acids and derivatives |
|  | 192.027 | C6H8O7 | Citric acid | M-H | Carboxylic acids and derivatives |
|  | 383.1077 | C14H17N5O8 | N6-Succinyladenosine | M-H | Purine nucleosides |
|  | 138.0317 | C7H6O3 | Gentisate aldehyde | M-H | Organooxygen compounds |
|  | 202.1317 | C9H18N2O3 | DL-Alanyl-DL-leucine | M-H | Carboxylic acids and derivatives |
|  | 260.1372 | C11H20N2O5 | Glutamylleucine | M-H | Carboxylic acids and derivatives |
|  | 184.0372 | C8H8O5 | 4-O-Methyl-gallate | M-H | Benzene and substituted derivatives |
|  | 138.0317 | C7H6O3 | Salicylic acid | M-H | Benzene and substituted derivatives |
|  | 354.0951 | C16H18O9 | Chlorogenic Acid | 2M-H | Organooxygen compounds |
|  | 230.163 | C11H22N2O3 | Leu-Val | M-H | Carboxylic acids and derivatives |
|  | 368.1107 | C17H20O9 | (1R,3R,4S,5R)-1,3,4-trihydroxy-5-[(E)-3-(4-hydroxy-3-methoxyphenyl)prop-2-enoyl]oxycyclohexane-1-carboxylic acid | M-H | Organooxygen compounds |
|  | 488.153 | C21H28O13 | NCGC00385695-01_C21H28O13_alpha-D-Glucopyranoside, beta-L-fructofuranosyl 6-O-[(2E)-3-(4-hydroxyphenyl)-1-oxo-2-propen-1-yl]- | M-H | Cinnamic acids and derivatives |
|  | 122.0368 | C7H6O2 | 4-Hydroxybenzaldehyde | M-H | Organooxygen compounds |
|  | 564.1479 | C26H28O14 | NP-000004(11) | M-H | Flavonoids |
|  | 158.0579 | C7H10O4 | 2-(methylethylidene)butanedioic acid | M-H | Fatty Acyls |
|  | 164.0473 | C9H8O3 | trans-P-Coumaric acid | M-H | Cinnamic acids and derivatives |
|  | 464.0955 | C21H20O12 | Quercetin-3-O-beta-D-galactoside | M-H | Flavonoids |
|  | 550.1686 | C26H30O13 | Liquiritin apioside | M-H | Flavonoids |
|  | 192.0423 | C10H8O4 | Scopoletin | M-H | Coumarins and derivatives |
|  | 478.1111 | C22H22O12 | Isorhamnetin 3-glucoside | M-H | Prenol lipids |
|  | 550.1686 | C26H30O13 | Liquiritin apioside | M-H | Flavonoids |
|  | 562.1686 | C27H30O13 | Isoflavone base + 1O, 1MeO, O-Pen-Hex | M-H | Isoflavonoids |
|  | 244.1311 | C12H20O5 | 4-Oxododecanedioic acid | M-H | Keto acids and derivatives |
|  | 264.1362 | C15H20O4 | (+)-Abscisic Acid | M-H | Prenol lipids |
|  | 284.0685 | C16H12O5 | Biochanin A | M-H | Isoflavonoids |
|  | 256.0736 | C15H12O4 | isoliquiritigenin | M-H | Linear 1,3-diarylpropanoids |
|  | 216.1362 | C11H20O4 | Undecanedioic acid | M-H | Fatty Acyls |
|  | 260.1988 | C14H28O4 | 3,12-Dihydroxytetradecanoic acid | M-H | Fatty Acyls |
|  | 822.4038 | C42H62O16 | Licoricesaponin H2 | M-H | Prenol lipids |
|  | 870.4613 | C44H70O17 | Furostane base -1H2O -2H + 1O, O-Hex, O-Pen-dHex | M+FA-H | Steroids and steroid derivatives |
|  | 822.4038 | C42H62O16 | Glycyrrhizin | M-H | Prenol lipids |
|  | 314.2457 | C18H34O4 | 12,13-DiHOME | M-H | Fatty Acyls |
|  | 596.2962 | C27H49O12P | LysoPI(18:2(9Z,12Z)/0:0) | M-H | Glycerophospholipids |
|  | 316.2614 | C18H36O4 | 9,10-Dihydroxystearic acid | M-H | Fatty Acyls |
|  | 432.2277 | C21H37O7P | LysoPA(18:3(9Z,12Z,15Z)/0:0) | M-H | Glycerophospholipids |
|  | 434.2433 | C21H39O7P | LysoPA(0:0/18:2(9Z,12Z)) | M-H | Glycerophospholipids |
|  | 286.24 | C15H10O6 | kaempferol | M-H | Flavonoids |
|  | 256.3 | C16H16O3 | Orchinol | M-H | Phenolic compounds |
|  | 174.1117 | C6H14N4O2 | L-Arginine monohydrochloride | M+H | Carboxylic acids and derivatives |
|  | 89.0477 | C3H7NO2 | beta-Alanine | M+H | Carboxylic acids and derivatives |
|  | 115.0633 | C5H9NO2 | Proline | M+H | Carboxylic acids and derivatives |
|  | 117.079 | C5H11NO2 | Betaine | M+H | Carboxylic acids and derivatives |
|  | 186.1004 | C8H14N2O3 | Alanylproline | M+H | Carboxylic acids and derivatives |
|  | 161.1052 | C7H15NO3 | Carnitine | M+H | Organonitrogen compounds |
|  | 115.0633 | C5H9NO2 | D-Pyrrolidine-2-carboxylic acid | M+H | Carboxylic acids and derivatives |
|  | 207.0895 | C11H13NO3 | N-Acetylphenylalanine | M+H | Carboxylic acids and derivatives |
|  | 383.1077 | C14H17N5O8 | N6-Succinyl adenosine | M+H | Purine nucleosides |
|  | 129.0426 | C5H7NO3 | L-Pyroglutamic acid | M+H | Carboxylic acids and derivatives |
|  | 327.1318 | C15H21NO7 | N-(1-Deoxy-1-fructosyl)phenylalanine | M+H | Carboxylic acids and derivatives |
|  | 157.0739 | C7H11NO3 | N-Acetylproline\|Acetyl Proline | M+H | Carboxylic acids and derivatives |
|  | 236.1161 | C12H16N2O3 | Alanylphenylalanine | M+H | Carboxylic acids and derivatives |
|  | 131.0946 | C6H13NO2 | Leucine | M+H | Carboxylic acids and derivatives |
|  | 205.0739 | C11H11NO3 | Dl-Indole-3-lactic acid | M+H-H2O | Indoles and derivatives |
|  | 302.24 | C15H10O7 | quercetin |  | Flavonoids |
|  | 266.1267 | C13H18N2O4 | Threonylphenylalanine | M+H | Carboxylic acids and derivatives |
|  | 204.0899 | C11H12N2O2 | Dl-Tryptophan | M+H | Indoles and derivatives |
|  | 161.0841 | C10H11NO | Tryptophanol | M+H-H2O | Indoles and derivatives |
|  | 564.1479 | C26H28O14 | NP-000062(6) | M+H | Flavonoids |
|  | 432.1056 | C21H20O10 | Isovitexin | M+H | Flavonoids\|Fatty Acyls |
|  | 448.1006 | C21H20O11 | Trifolin | M+H | Pyrans |
|  | 313.35 | C18H19NO4 | N-Trans-Feruloyltyramine |  |  |
|  | 432.1056 | C21H20O10 | Apigetrin | M+H | Flavonoids |
|  | 418.1264 | C21H22O9 | Liquiritin | M+H | Flavonoids |
|  | 256.0736 | C15H12O4 | Isoliquiritigenin | M+H | Linear 1,3-diarylpropanoids |
|  | 418.1264 | C21H22O9 | 2-(4-hydroxyphenyl)-7-[(2S,3R,4S,5S,6R)-3,4,5-trihydroxy-6-(hydroxymethyl)oxan-2-yl]oxy-2,3-dihydrochromen-4-one | M+H | Flavonoids |
|  | 430.1264 | C22H22O9 | Ononin | M+H | Isoflavonoids |
|  | 594.1373 | C30H26O13 | Kaempferol-3-Glucoside-6''-p-coumaroyl | M+H | Flavonoids |
|  | 284.0685 | C16H12O5 | Calycosin | M+H | Isoflavonoids |
|  | 446.1213 | C22H22O10 | Sissotrin | M+H | Isoflavonoids |
|  | 310.2144 | C18H30O4 | 9(S)-HpOTrE | M+H-H2O | Fatty Acyls |
|  | 268.0736 | C16H12O4 | 7-hydroxy-3-(4-methoxyphenyl)chromen-4-one | M+H | Isoflavonoids |
|  | 276.2089 | C18H28O2 | Stearidonic acid | M+H | Fatty Acyls |
|  | 822.4038 | C42H62O16 | Glycyrrhizate | M+H | Prenol lipids |
|  | 432.2148 | C24H32O7 | Schisandrin | M+H | Tannins |
|  | 296.2351 | C18H32O3 | 13(S)-HODE\|13-HODE | M+H-H2O | Fatty Acyls |
|  | 500.241 | C28H36O8 | Angeloylgomisin H | M+Na | Tannins |
|  | 317.293 | C18H39NO3 | Phytosphingosine | M+H | Organonitrogen compounds |
|  | 514.2203 | C28H34O9 | Schisanwilsonin I | M+Na | Tannins |
|  | 294.2195 | C18H30O3 | 13(S)-HOTrE | M+H-H2O | Fatty Acyls |
|  | 294.2195 | C18H30O3 | 9-Oxoode | M+H | Fatty Acyls |
|  | 400.1886 | C23H28O6 | Rubschisandrin | M+H | Tannins |
