# Supplementary material for: Combined Lingzhi Huang capsules and Zeng Jian health tonic accelerates skin wound healing via BMP5-mediated inhibition of ferroptosis
Source: Front Immunol. 2026 Jun 2;17:1818752. doi: 10.3389/fimmu.2026.1818752 (PMC13268888; doi:10.3389/fimmu.2026.1818752)
Supplement: Supplementary file 2 [file DataSheet2.docx]

**TableS2. LC-MS/MS data of characterized compounds in ZJ**

| No. | Molecular weight (g/mol) | Formula | Compounds | Ion mode | Class |
| --- | --- | --- | --- | --- | --- |
|  | 272.25 | C15H12O5 | (R)-naringenin | M-H | Flavonoids |
|  | 146.0579 | C6H10O4 | Adipic acid | M-H | Fatty Acyls |
|  | 205.0739 | C11H11NO3 | Indole-3-lactic acid | M-H | Indoles and derivatives |
|  | 592.1792 | C28H32O14 | Acacetin-7-O-rutinoside | M+FA-H | Flavonoids |
|  | 354.0951 | C16H18O9 | (3R,5S)-4-[(E)-3-(3,4-dihydroxyphenyl)prop-2-enoyl]oxy-1,3,5-trihydroxycyclohexane-1-carboxylic acid | M+H | Organooxygen compounds |
|  | 354.0951 | C16H18O9 | 5-Caffeoylquinic acid | M+H | Organooxygen compounds |
|  | 86.0368 | C4H6O2 | Gamma-Butyrolactone | M+H | Lactones |
|  | 269.3083 | C18H39N | 1-Octadecanamine | M+H | Organonitrogen compounds |
|  | 145.0739 | C6H11NO3 | 4-Acetamidobutyric acid | M+H | Carboxylic acids and derivatives |
|  | 133.0375 | C4H7NO4 | Aspartic acid | M-H | Carboxylic acids and derivatives |
|  | 146.0579 | C6H10O4 | 3-Methylglutaric acid | M-H | Fatty Acyls |
|  | 354.0951 | C16H18O9 | (3R,5R)-1-[(E)-3-(3,4-dihydroxyphenyl)prop-2-enoyl]oxy-3,4,5-trihydroxycyclohexane-1-carboxylic acid | M+H | Organooxygen compounds |
|  | 244.1311 | C12H20O5 | 4-Oxododecanedioic acid | M-H | Keto acids and derivatives |
|  | 608.1741 | C28H32O15 | 5-hydroxy-2-(3-hydroxy-4-methoxyphenyl)-7-[3,4,5-trihydroxy-6-[(3,4,5-trihydroxy-6-methyloxan-2-yl)oxymethyl]oxan-2-yl]oxychromen-4-one | M-H | Flavonoids |
|  | 122.0368 | C7H6O2 | 4-Hydroxybenzaldehyde | M-H | Organooxygen compounds |
|  | 164.0473 | C9H8O3 | trans-P-Coumaric acid | M-H | Cinnamic acids and derivatives |
|  | 595.1663 | [C27H31O15]+ | pelargonidin-3,5-di-O-glucoside | M | Flavonoids |
|  | 147.0532 | C5H9NO4 | Glutamic acid | M-H | Carboxylic acids and derivatives |
|  | 354.0951 | C16H18O9 | Chlorogenic Acid | 2M-H | Organooxygen compounds |
|  | 160.0736 | C7H12O4 | Pimelic acid | M-H | Fatty Acyls |
|  | 578.1636 | C27H30O14 | Apigenin 7-O-neohesperidoside | M+H | Flavonoids |
|  | 607.0816 | C17H27N3O17P2 | Uridine diphosphate-N-acetylglucosamine | M-H | Pyrimidine nucleotides |
|  | 594.1585 | C27H30O15 | Kaempferol-7-O-neohesperidoside | M-H | Flavonoids |
|  | 189.0426 | C10H7NO3 | Kynurenic acid | M-H | Quinolines and derivatives |
|  | 592.1792 | C28H32O14 | Linarin | M+H | Organooxygen compounds\|Flavonoids |
|  | 152.0685 | C5H12O5 | Xylitol | M-H | Organooxygen compounds |
|  | 284.0685 | C16H12O5 | Acacetin | M+H | Flavonoids |
|  | 300.0634 | C16H12O6 | Diosmetin | M-H | Flavonoids |
|  | 286.0477 | C15H10O6 | Luteolin | 2M-H | Flavonoids |
|  | 432.1056 | C21H20O10 | Apigetrin | M+H | Flavonoids |
|  | 518.106 | C24H22O13 | Isoflavone base + 3O, O-MalonylHex | M+H | Isoflavonoids |
|  | 196.0583 | C6H12O7 | Gluconic acid | M-H | Organooxygen compounds\|Hydroxy acids and derivatives |
|  | 99.0684 | C5H9NO | 2-Piperidone | M+H | Piperidines |
|  | 344.0896 | C18H16O7 | 5,7-dihydroxy-3,6-dimethoxy-2-(4-methoxyphenyl)-4H-chromen-4-one | 2M+Na | Flavonoids |
|  | 432.1056 | C21H20O10 | Genistin | M+H | Isoflavonoids |
|  | 284.0685 | C16H12O5 | Acacetin | M+H | Flavonoids |
|  | 202.1317 | C9H18N2O3 | L-Leucyl-L- Alanine | M+H | Carboxylic acids and derivatives |
|  | 478.0747 | C21H18O13 | Quercetin 3-O-glucuronide | M+H | Flavonoids |
|  | 460.1006 | C22H20O11 | Oroxindin | M+H | Flavonoids |
|  | 149.051 | C5H11NO2S | Racemethionine | M+H | Carboxylic acids and derivatives |
|  | 88.016 | C3H4O3 | Pyruvic acid | M-H | Keto acids and derivatives |
|  | 219.1107 | C9H17NO5 | 3-[[(2S)-2,4-Dihydroxy-3,3-dimethylbutanoyl]amino]propanoic acid | M+H | Carboxylic acids and derivatives |
|  | 478.1111 | C22H22O12 | Isorhamnetin-3-glucoside | M+H | Prenol lipids |
|  | 530.3104 | C28H42N4O6 | Kukoamine B | M+H | Phenols |
|  | 516.1268 | C25H24O12 | (3R,5R)-3,5-bis[[(E)-3-(3,4-dihydroxyphenyl)prop-2-enoyl]oxy]-1,4-dihydroxycyclohexane-1-carboxylic acid | M-H2O+H | Organooxygen compounds |
|  | 270.0528 | C15H10O5 | 1,10-dihydroxy-3-methylbenzo[c][1]benzoxepine-6,11-dione | M+H | Benzoxepines |
|  | 383.1077 | C14H17N5O8 | N6-Succinyl adenosine | M+H | Purine nucleosides |
|  | 192.0634 | C7H12O6 | Quinate | M-H | Organooxygen compounds |
|  | 264.1362 | C15H20O4 | (+)-Abscisic Acid | M-H | Prenol lipids |
|  | 276.2089 | C18H28O2 | Stearidonic acid | M+H | Fatty Acyls |
|  | 157.0739 | C7H11NO3 | N-Acetylproline | M+H | Carboxylic acids and derivatives |
|  | 129.0426 | C5H7NO3 | 5-Oxo-L-Proline | M+H | Carboxylic acids and derivatives |
|  | 230.163 | C11H22N2O3 | Valylleucine | M+H-H2O | Carboxylic acids and derivatives |
|  | 117.079 | C5H11NO2 | Betaine | M+H | Carboxylic acids and derivatives |
|  | 138.0317 | C7H6O3 | Salicylic acid | M-H | Benzene and substituted derivatives |
|  | 316.0583 | C16H12O7 | Isorhamnetin | M-H | Flavonoids |
|  | 220.1827 | C15H24O | Farnesal | M+H-H2O | Prenol lipids |
|  | 196.0583 | C6H12O7 | D-Galactonic acid | M-H | Hydroxy acids and derivatives |
|  | 255.2562 | C16H33NO | Palmitamide | M+H | Fatty Acyls |
|  | 284.0685 | C16H12O5 | Xenognosin B | M-H | Isoflavonoids |
|  | 103.0997 | C5H13NO | Choline | M+H | Organonitrogen compounds |
|  | 181.0739 | C9H11NO3 | D-Tyrosine | M+H | Carboxylic acids and derivatives |
|  | 300.0634 | C16H12O6 | Xanthorin | M-H | Anthracenes |
|  | 516.1268 | C25H24O12 | 1,4-Dicaffeoylquinic acid | M-H | Organooxygen compounds |
|  | 89.0477 | C3H7NO2 | L-Alanine | M+H | Carboxylic acids and derivatives |
|  | 432.1056 | C21H20O10 | Apigenin-8-C-glucoside | M+H | Flavonoids |
|  | 192.1514 | C13H20O | beta-Lonone | M+H-H2O | Prenol lipids |
|  | 103.0633 | C4H9NO2 | Kaempferol-3-Glucoside-3''-Rhamnoside |  | Carboxylic acids and derivatives |
|  | 300.0634 | C16H12O6 | Chrysoeriol | M-H | Flavonoids |
|  | 374.1002 | C19H18O8 | Chrysosplenetin | M+H | Flavonoids |
|  | 272.2351 | C16H32O3 | 2-hydroxyhexadecanoic acid | M-H | Fatty Acyls |
|  | 228.1362 | C12H20O4 | Traumatic acid | M+H | Fatty Acyls |
|  | 178.0266 | C9H6O4 | Esculetin | M+H | Coumarins and derivatives |
|  | 474.1162 | C23H22O11 | Apigenin 7-O-(6''-O-acetylglucoside) | M+H | Flavonoids |
|  | 210.1368 | C11H18N2O2 | Cyclo(proline-leucine) | M+H | Carboxylic acids and derivatives |
|  | 478.1111 | C22H22O12 | Isorhamnetin 3-glucoside | M-H | Prenol lipids |
|  | 205.0739 | C11H11NO3 | Dl-Indole-3-lactic acid | M+H-H2O | Indoles and derivatives |
|  | 464.0955 | C21H20O12 | 3,5,7-trihydroxy-2-[4-hydroxy-3-[(2S,3R,4S,5S,6R)-3,4,5-trihydroxy-6-(hydroxymethyl)oxan-2-yl]oxyphenyl]chromen-4-one | M+FA-H | Flavonoids |
|  | 180.0423 | C9H8O4 | Caffeic acid | M+H-H2O | Cinnamic acids and derivatives |
|  | 286.0477 | C15H10O6 | Kaempferol | M-H | Flavonoids |
