# Supplementary material for: Combined Lingzhi Huang capsules and Zeng Jian health tonic accelerates skin wound healing via BMP5-mediated inhibition of ferroptosis
Source: Front Immunol. 2026 Jun 2;17:1818752. doi: 10.3389/fimmu.2026.1818752 (PMC13268888; doi:10.3389/fimmu.2026.1818752)
Supplement: Supplementary file 3 [file Supplementaryfile1.docx]

Supplementary Materials


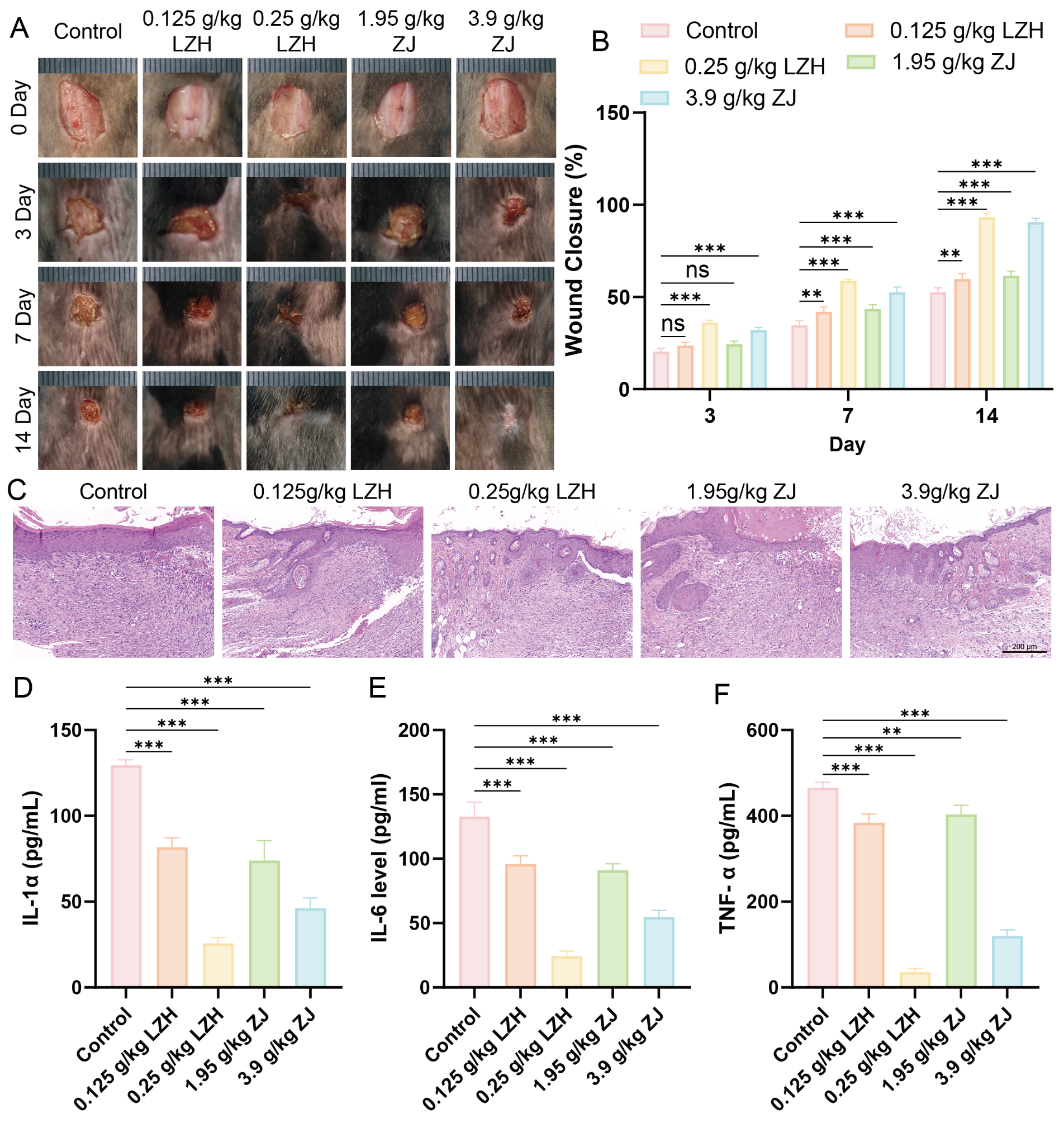


**Figure S1.** (A) Representative wound photographs and mode patterns from control, 0.125 g/kg LZH, 0.25 g/kg LZH, 1.95 g/kg ZJ, and 3.9 g/kg ZJ groups on days 0, 3, 7, and 14. (B) The wound closure was quantified by calculating the percentage of the wound area relative to that measured on day 0. (n = 3). (C) Representative H&E staining images of wound tissues on days 14. Scale bar = 200 μm. (D-F) ELISA analysis of IL-1α, IL-6, and TNF-α levels in different treatment groups (n = 3). Data is presented as mean ± SD. *P*-values are calculated using one-way analysis of variance (ANOVA) or two-way analysis of variance (ANOVA) for multiple group comparisons. ^*^*P* < 0.05, ^**^*P* < 0.01, ^***^*P* < 0.001, ns, not significant.


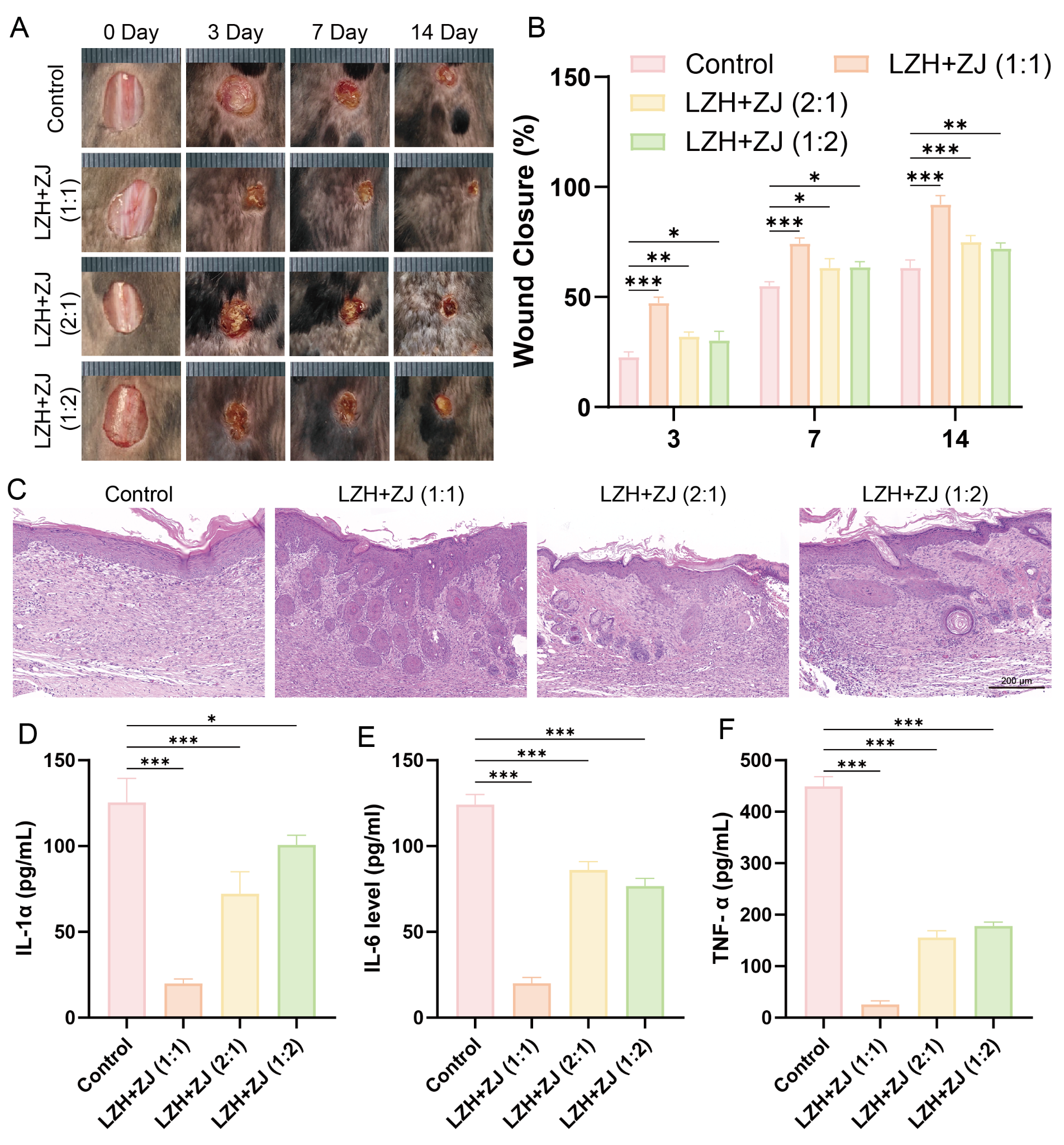


**Figure S2.** (A) Representative wound photographs and mode patterns from control, LZH+ZJ (1:1), LZH+ZJ (2:1), LZH+ZJ (1:2) groups on days 0, 3, 7, and 14. (B) The wound closure was quantified by calculating the percentage of the wound area relative to that measured on day 0. (n = 3). (C) Representative H&E staining images of wound tissues on days 14. Scale bar = 200 μm. (D-F) ELISA analysis of IL-1α, IL-6, and TNF-α levels in different treatment groups (n = 3). Data is presented as mean ± SD. *P*-values are calculated using one-way analysis of variance (ANOVA) or two-way analysis of variance (ANOVA) for multiple group comparisons. ^*^*P* < 0.05, ^**^*P* < 0.01, ^***^*P* < 0.001.


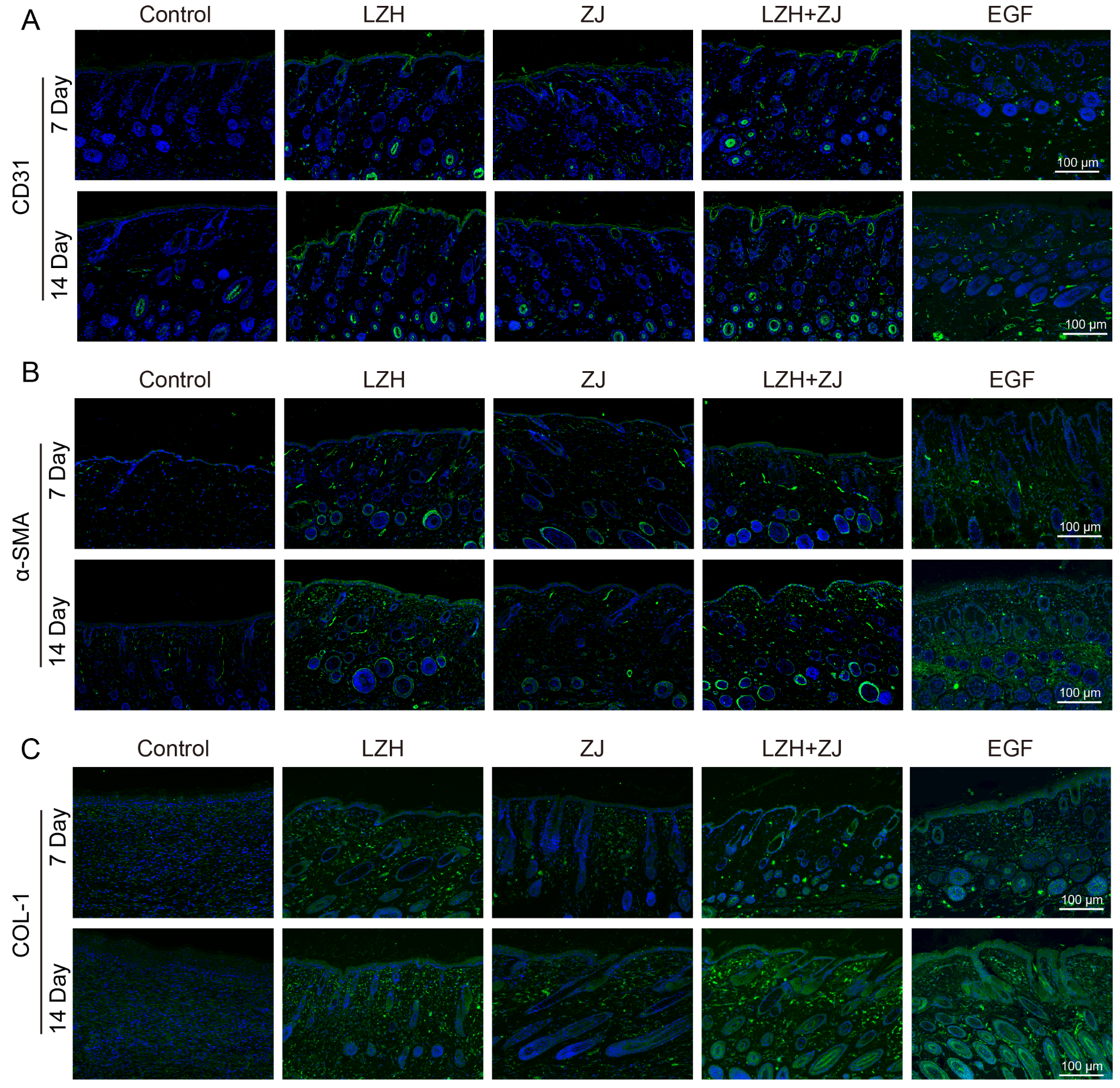


**Figure S3.** (A-C) Representative immunofluorescence images showing the expression of CD31, α-SMA, and COL-1 in wound sections from different groups at 7 and 14 days. Scale bars = 100 μm. (n = 5).


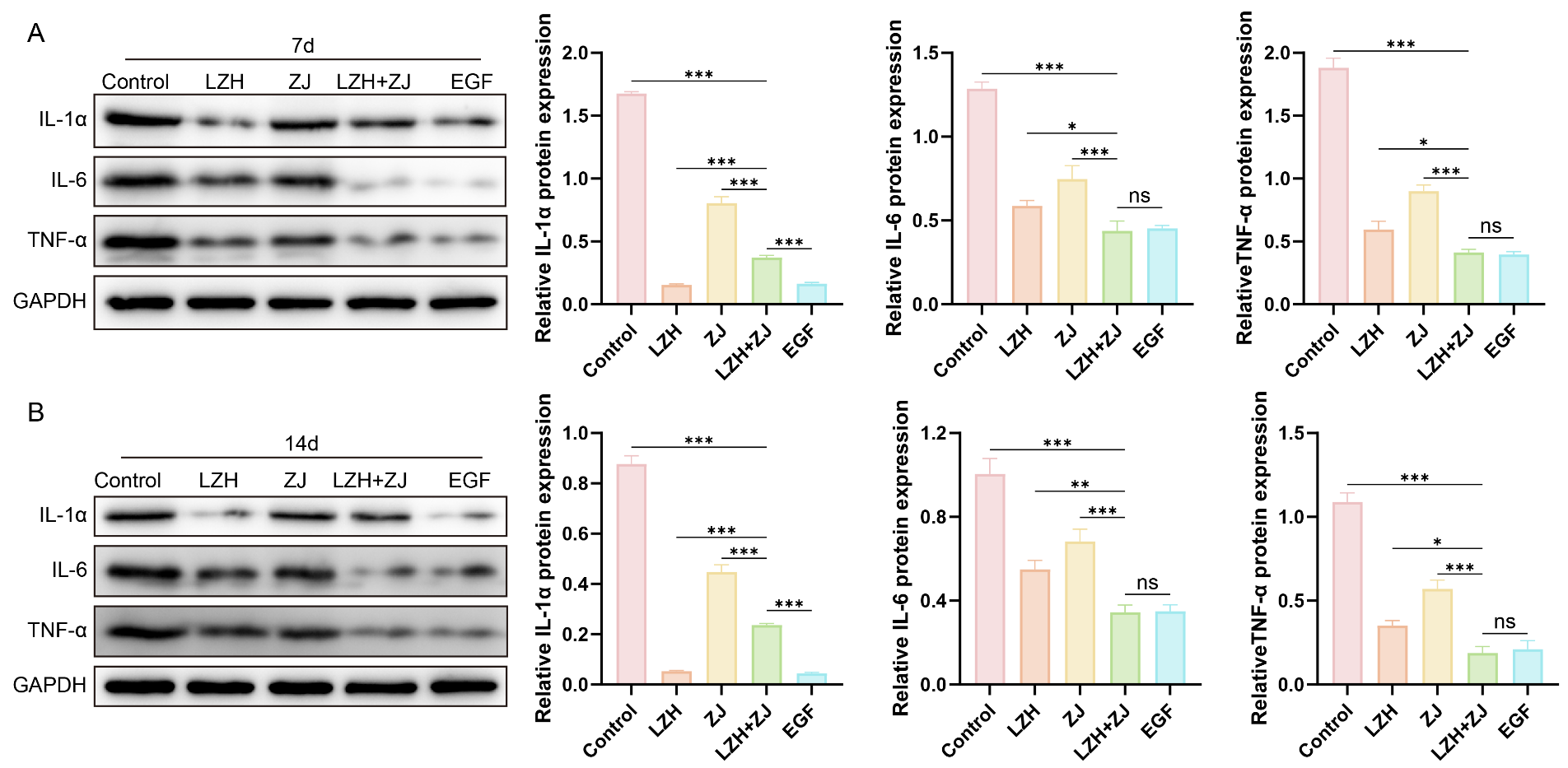


**Figure S4.** (A-B) Western blot analysis of IL-1α, IL-6, and TNF-α levels in wound tissues at 7 and 14 days. (n = 3). Data is presented as mean ± SD. *P*-values are calculated using one-way analysis of variance (ANOVA) for multiple group comparisons. ^*^*P* < 0.05, ^**^*P* < 0.01, ^***^*P* < 0.001, ns, not significant.


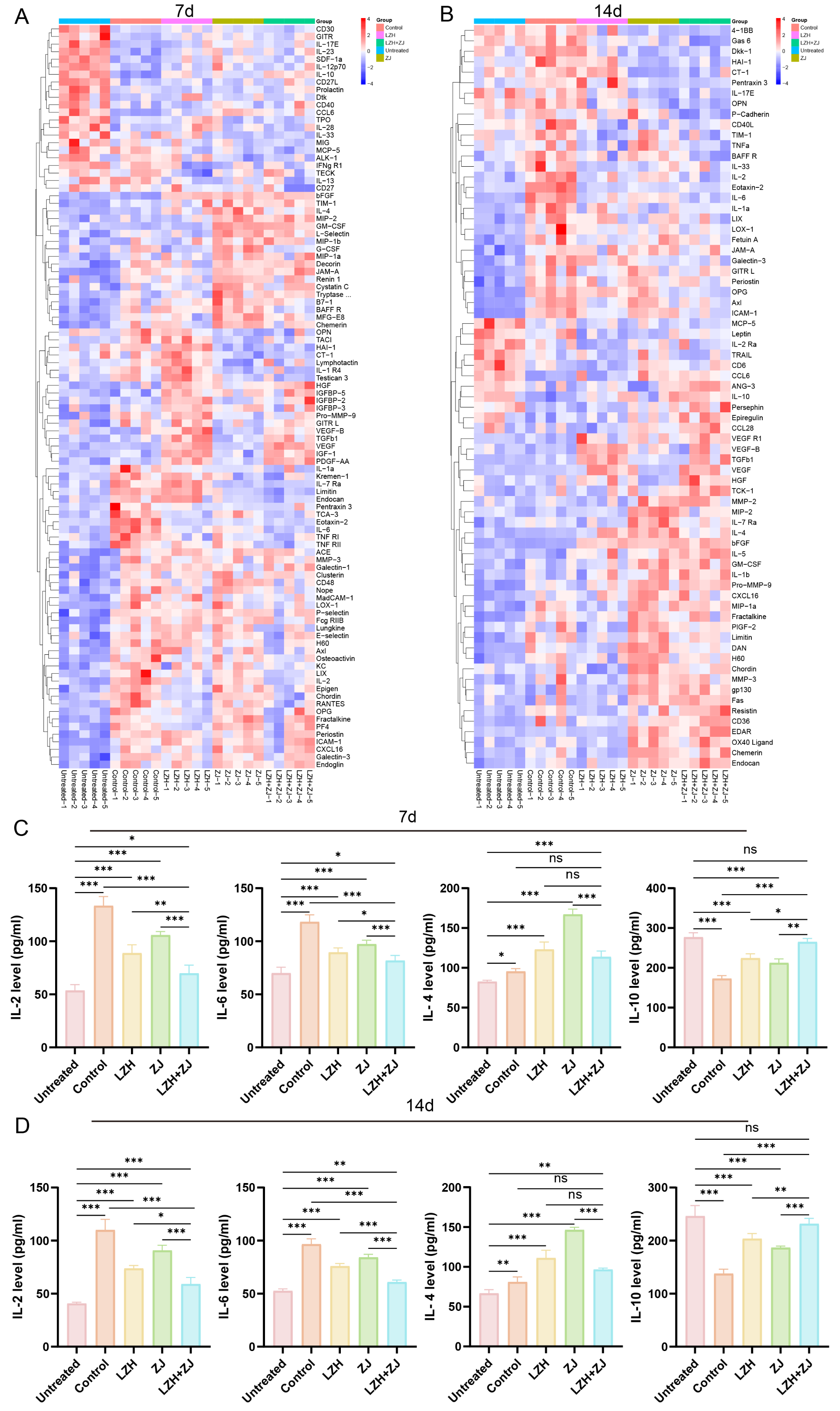


**Figure S5.** (A-B) Heatmaps showing the alterations in multiple factors in skin wound tissues from different groups at days 7 and 14. (C-D) Serum levels of IL-2, IL-6, IL-4, and IL-10 on days 7 and 14 were measured using ELISA. (n = 5). Data is presented as mean ± SD. *P*-values are calculated using one-way analysis of variance (ANOVA) for multiple group comparisons. ^*^*P* < 0.05, ^**^*P* < 0.01, ^***^*P* < 0.001, ns, not significant.


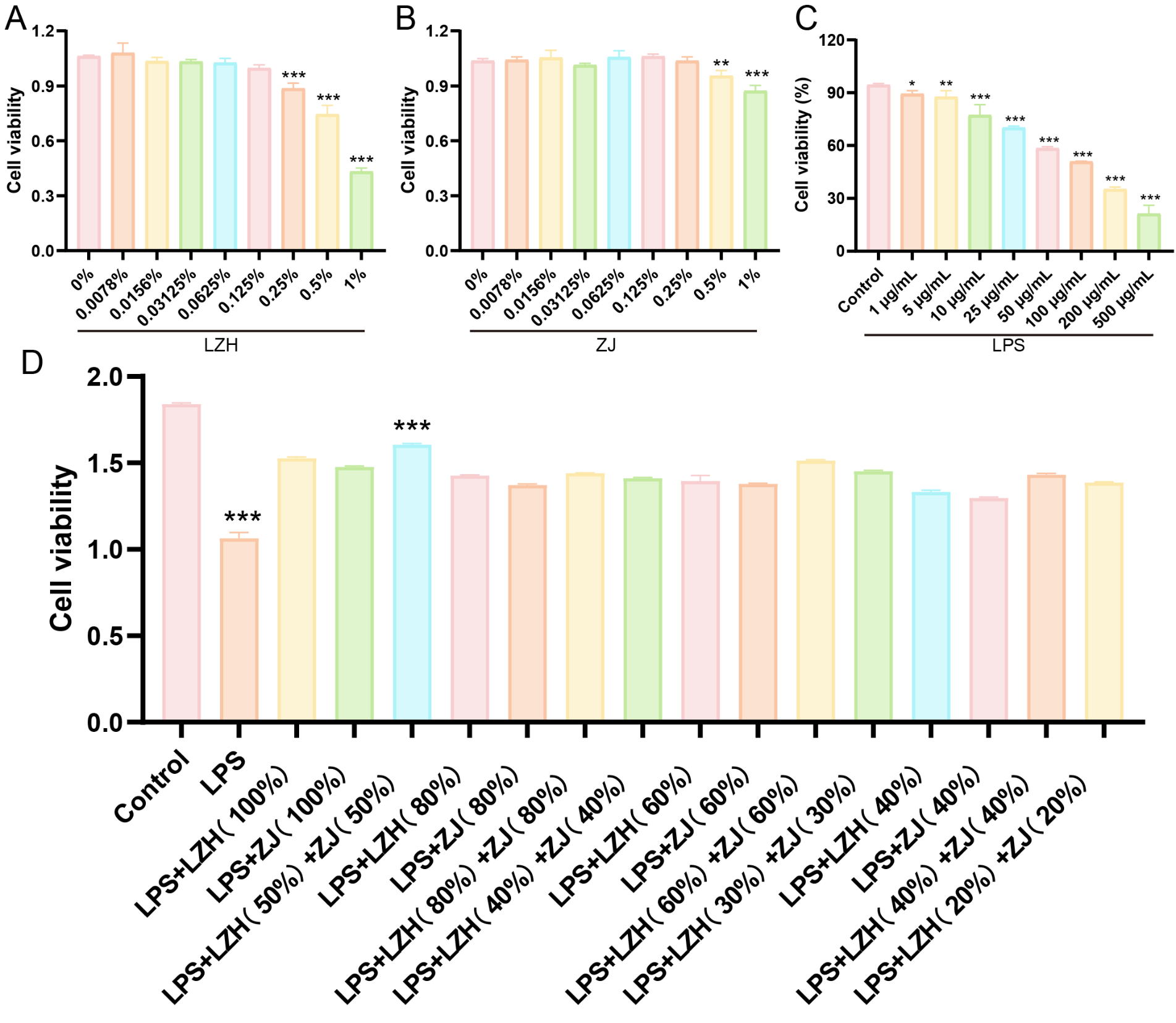


**Figure S6.** (A) CCK-8 assay showing cell viability following exposure to varying concentrations of LZH. (n = 3). (B) CCK-8 assay showing cell viability following exposure to varying concentrations of ZJ. (n = 3). (C) CCK-8 assay showing cell viability following exposure to varying concentrations of LPS. (n = 3). (D) CCK-8 assay showing cell viability following exposure to varying concentrations of combined LZH and ZJ. (n = 3). Data is presented as mean ± SD. *P*-values are calculated using one-way analysis of variance (ANOVA) for multiple group comparisons. ^*^*P* < 0.05, ^**^*P* < 0.01, ^***^*P* < 0.001.


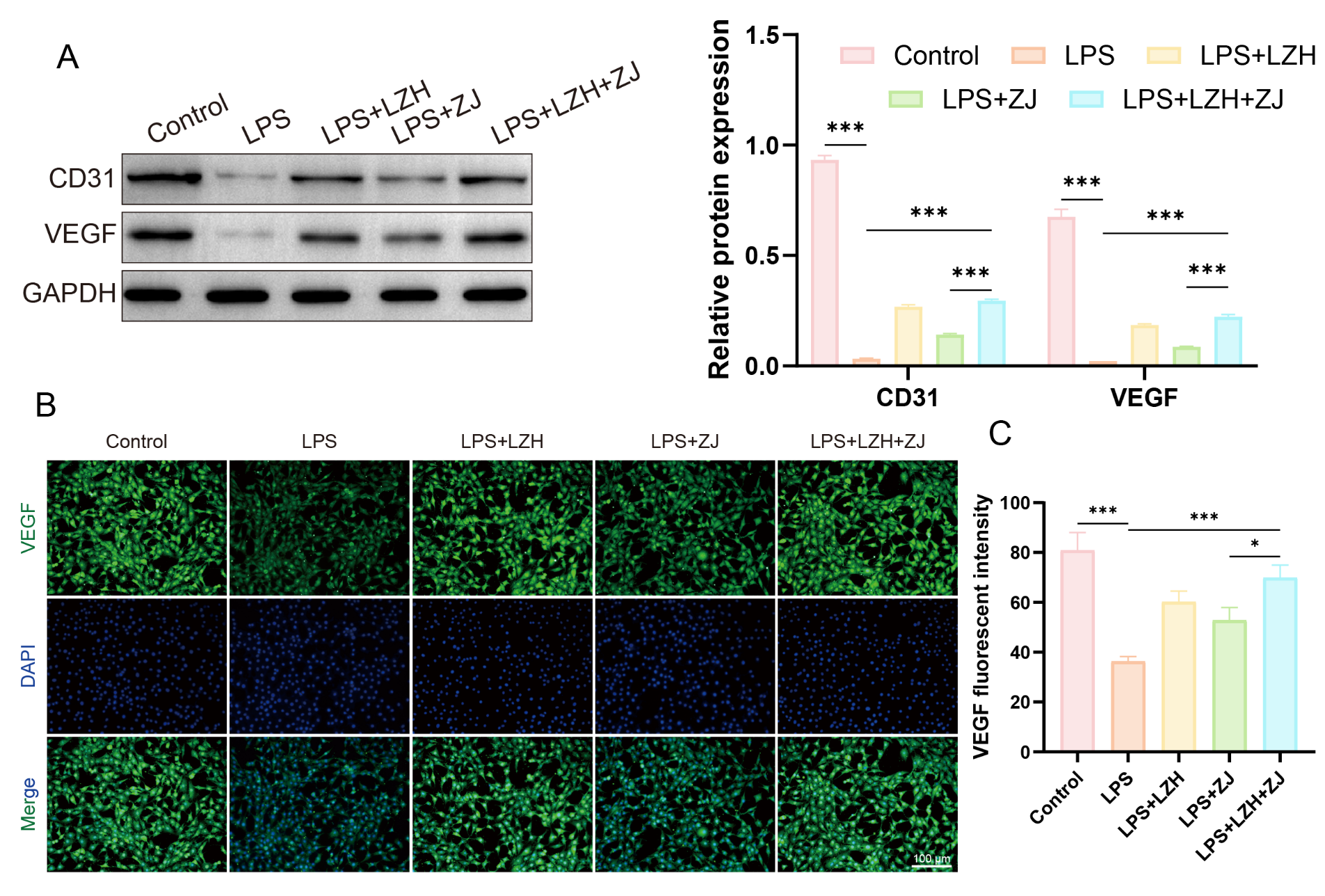


**Figure S7.** (A) Western blot analysis of CD31 and VEGF protein expression in different treatment groups. (n = 3). (B-C) Immunofluorescence staining and quantitative analysis of VEGF. Scale bar = 100 µm. (n = 3). Data is presented as mean ± SD. *P*-values are calculated using one-way analysis of variance (ANOVA) or two-way analysis of variance (ANOVA) for multiple group comparisons. ^*^*P* < 0.05, ^**^*P* < 0.01, ^***^*P* < 0.001.


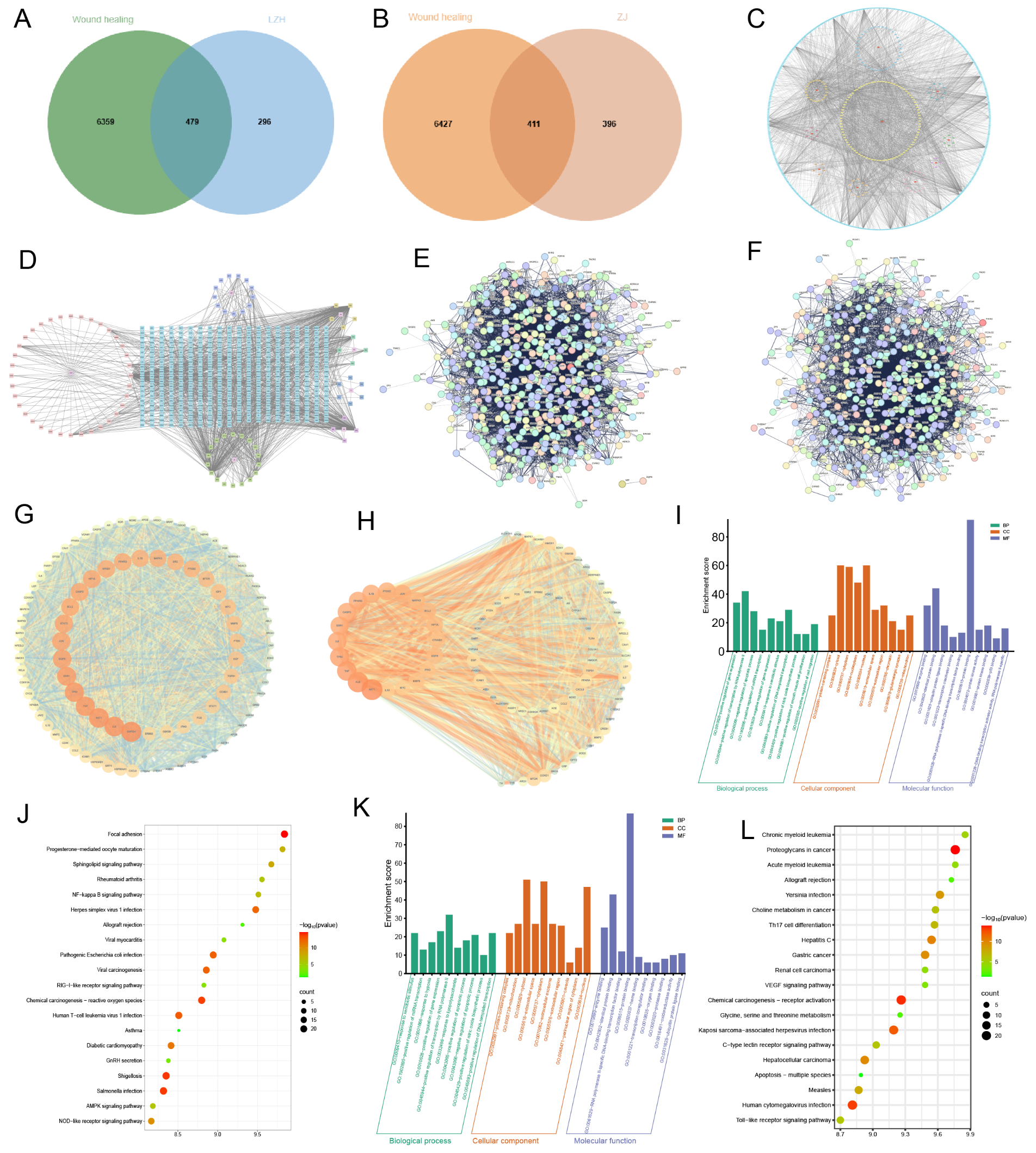


**Figure S8.** (A-B) Venn diagrams showing the targets of LZH-wound healing and ZJ-wound healing. (C-D) Drug-component-target networks of LZH-wound healing and ZJ-wound healing. (E-F) PPI networks of LZH-wound healing and ZJ-wound healing. (G-H) Core target maps of LZH-wound healing and ZJ-wound healing. (I-L) GO and KEGG enrichment analyses results for LZH-wound healing and ZJ-wound healing.


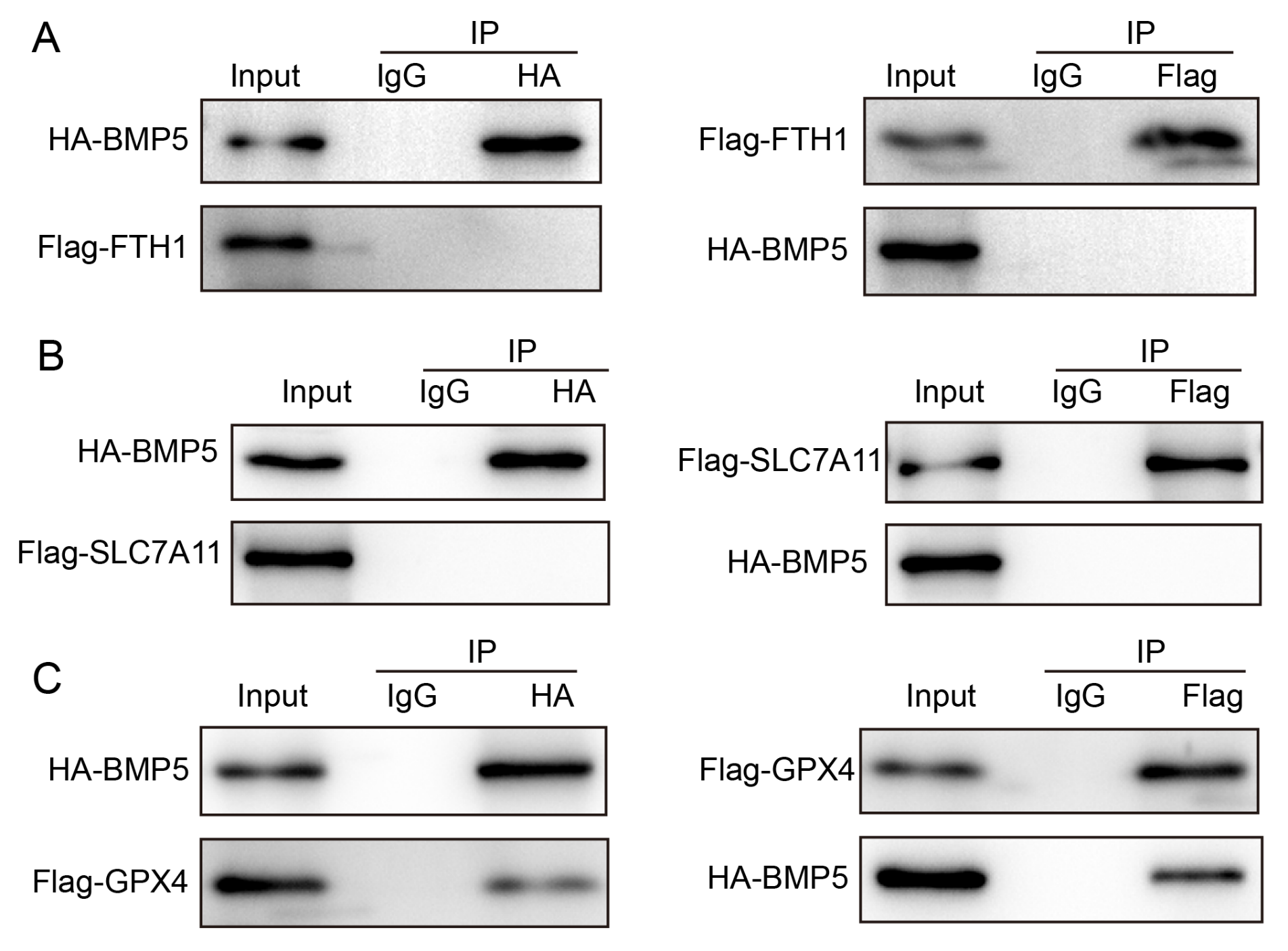


**Figure S9.** (A-C) Co-immunoprecipitation assays showing BMP5 interaction with FTH1, SLC7A11, and GPX4 in 293T cells. (n = 3).


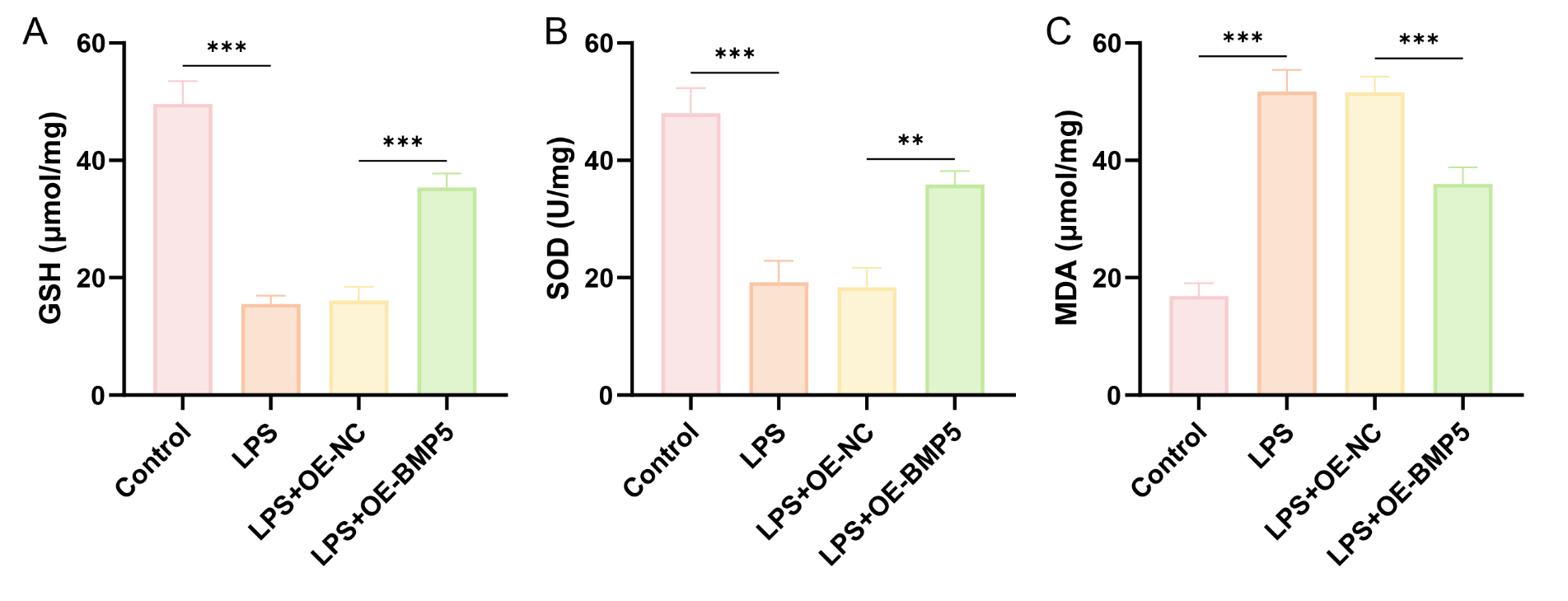


**Figure S10.** (A-C) The levels of GSH, SOD, and MDA were quantified using ELISA. (n = 3). Data is presented as mean ± SD. *P*-values are calculated using one-way analysis of variance (ANOVA) for multiple group comparisons. ^*^*P* < 0.05, ^**^*P* < 0.01, ^***^*P* < 0.001.
